# *Bordetella* filamentous hemagglutinin and adenylate cyclase toxin interactions on the bacterial surface are consistent with FhaB-mediated delivery of ACT to phagocytic cells

**DOI:** 10.1128/mbio.00632-24

**Published:** 2024-03-27

**Authors:** Zachary M. Nash, Carol S. Inatsuka, Peggy A. Cotter, Richard M. Johnson

**Affiliations:** 1 Department of Microbiology and Immunology, School of Medicine, University of North Carolina-Chapel Hill, Chapel Hill, North Carolina, USA; 2 Department of Molecular, Cellular and Developmental Biology, University of California, Santa Barbara, Santa Barbara, California, USA; New York University School of Medicine, New York, New York, USA

**Keywords:** *Bordetella*, filamentous hemagglutinin, Type V secretion, RTX toxins, adenylate cyclase, protein-protein interactions

## Abstract

*Bordetella* species that cause respiratory infections in mammals include *B. pertussis*, which causes human whooping cough, and *B. bronchiseptica*, which infects nearly all mammals. Both bacterial species produce filamentous hemagglutinin (FhaB) and adenylate cyclase toxin (ACT), prominent surface-associated and secreted virulence factors that contribute to persistence in the lower respiratory tract by inhibiting clearance by phagocytic cells. FhaB and ACT proteins interact with themselves, each other, and host cells. Using immunoblot analyses, we showed that ACT binds to FhaB on the bacterial surface before it can be detected in culture supernatants. We determined that SphB1, a surface protease identified based on its requirement for FhaB cleavage, is also required for ACT cleavage, and we determined that the presence of ACT blocks SphB1-dependent and -independent cleavage of FhaB, but the presence of FhaB does not affect SphB1-dependent cleavage of ACT. The primary SphB1-dependent cleavage site on ACT is proximal to ACT’s active site, in a region that is critical for ACT activity. We also determined that FhaB-bound ACT on the bacterial surface can intoxicate host cells producing CR3, the receptor for ACT. In addition to increasing our understanding of FhaB, ACT, and FhaB-ACT interactions on the *Bordetella* surface, our data are consistent with a model in which FhaB functions as a novel toxin delivery system by binding to ACT and allowing its release upon binding of ACT to its receptor, CR3, on phagocytic cells.

**IMPORTANCE:**

Bacteria need to control the variety, abundance, and conformation of proteins on their surface to survive. Members of the Gram-negative bacterial genus *Bordetella* include *B. pertussis*, which causes whooping cough in humans, and *B. bronchiseptica*, which causes respiratory infections in a broad range of mammals. These species produce two prominent virulence factors, the two-partner secretion (TPS) effector FhaB and adenylate cyclase toxin (ACT), that interact with themselves, each other, and host cells. Here, we determined that ACT binds FhaB on the bacterial surface before being detected in culture supernatants and that ACT bound to FhaB can be delivered to eukaryotic cells. Our data are consistent with a model in which FhaB delivers ACT specifically to phagocytic cells. This is the first report of a TPS system facilitating the delivery of a separate polypeptide toxin to target cells and expands our understanding of how TPS systems contribute to bacterial pathogenesis.

## INTRODUCTION

Interactions between proteins on a bacterial surface with each other or with biotic or abiotic surfaces often have consequences that are critical to bacterial survival. Such interactions can mediate adherence of the bacteria to cells or surfaces and facilitate the delivery of proteins, including toxins, to nearby cells. *Bordetella pertussis*, the causal agent of human whooping cough, and the closely related species *Bordetella bronchiseptica*, which causes respiratory infections in a broad range of mammals, produce two prominent virulence factors, filamentous hemagglutinin (FhaB) and adenylate cyclase toxin (ACT), that interact with themselves, each other (1), and molecules on host cells. Although both FhaB and ACT play roles in adherence (1–5), biofilm formation (6, 7), and persistence in the lower respiratory tract (8–11), the mechanisms and importance of FhaB-ACT interactions in mediating these phenotypes are not well understood.

**Fig 1:**
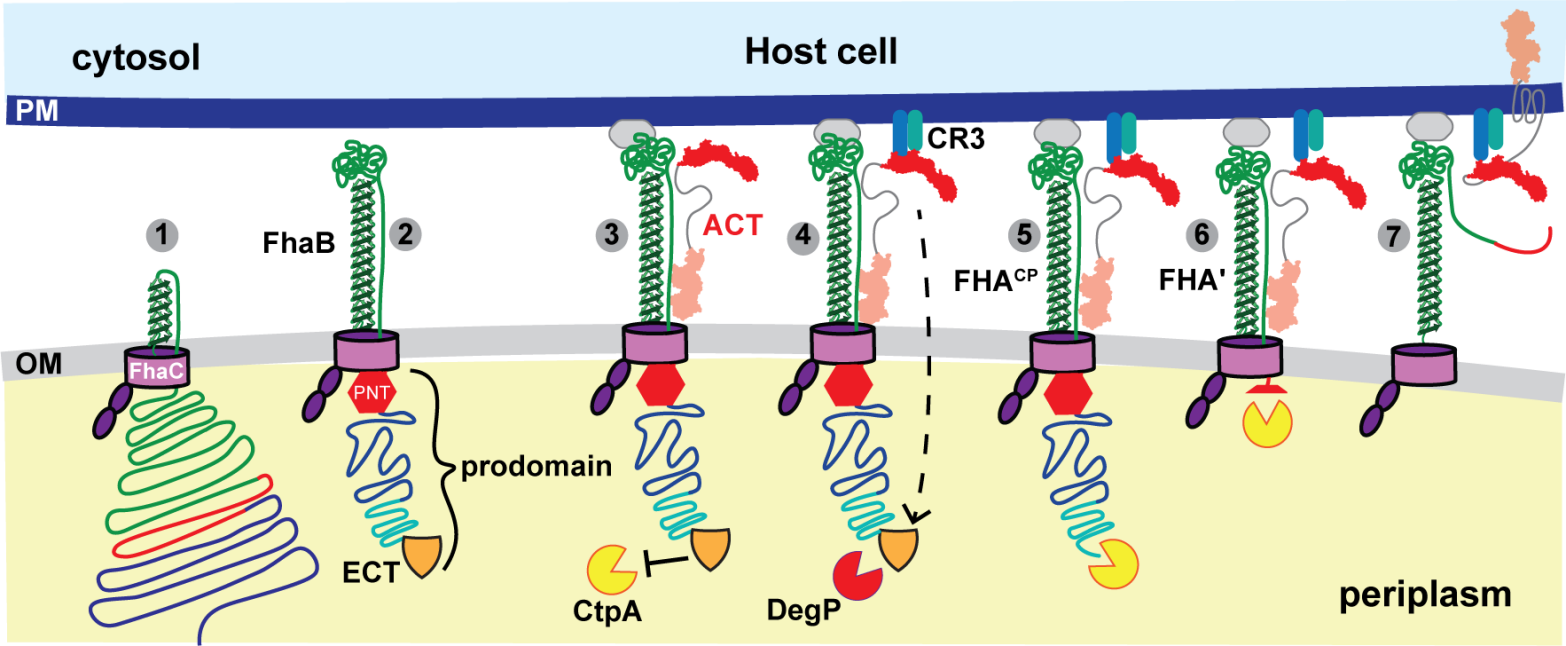
Hypothetical model for FhaB-mediated delivery of ACT to CR3+ cells. FhaB is secreted across the outer membrane (OM) by FhaC until the FhaB prodomain N-terminus (PNT, red stop sign) arrests secretion and stabilizes the formation of a hairpin on the cell surface (steps 1–2). The N-terminal adenylate cyclase domain of ACT binds to the FhaB mature C-terminal domain forming a stable complex (step 3). Engagement of FhaB with an unknown receptor and ACT with its receptor (CR3) causes a signal to be transduced that initiates regulated degradation of the periplasmic FhaB prodomain by DegP and CtpA (steps 4–6). Prodomain degradation begins with the removal of the FhaB extreme C terminus (ECT, orange shield, step 4) and causes the C terminus of processed FhaB to exit the FhaC channel culminating in delivery of ACT to the CR3+ host cell and release of FHA′ (step 7).

FhaB and its outer membrane transporter FhaC compose the prototypical two-partner secretion (TPS, also known as Type Vb) system, a family of bacterial secretion systems that is broadly distributed among Gram-negative bacteria (12–14). Decades of research using *B. pertussis* or *B. bronchiseptica* grown in Stainer Scholte (SS) broth (15) or on Bordet-Gengou (BG) agar (16), or using *Escherichia coli* producing all or part of FhaB and FhaC, have led to the model of FhaB secretion shown in Fig. 1. FhaB is synthesized initially as a ~375 kD preproprotein with an extended N-terminal signal sequence that mediates Sec-dependent delivery to the periplasm (17, 18), and a TPS domain that interacts with FhaC’s periplasmic POTRA domains to initiate translocation across the outer membrane (19–21) (not shown in Fig. 1). FhaB then emerges on the bacterial surface as a hairpin with its N terminus anchored to FhaC while approximately two-thirds of FhaB are translocated through the FhaC barrel in the N- to C-terminal direction, forming a β-helical shaft topped with a globular “mature C-terminal domain” (MCD) (Fig. 1 steps 1 and 2) (22). A ~200 amino acid (aa) region, called the prodomain N terminus (PNT, Fig. 1), forms a molecular knot that blocks translocation through FhaC such that the remaining C-terminal ~1,200 aa of FhaB, called the prodomain, are retained in the periplasm (Fig. 1, step 2) (23). Full-length FhaB forms a stable complex with FhaC, with the extreme C terminus (ECT, represented as a shield in Fig. 1) protecting the prodomain from degradation. ACT (described below) is secreted *via* a dedicated Type I secretion system (not shown) and binds FhaB (Fig. 1, step 3). We hypothesize that the FhaC-FhaB-ACT complex remains stably associated on the bacterial surface until regulated processing of the prodomain begins with a signal that promotes DegP-dependent removal of the ECT to form FhaB^CP^ (Fig. 1 step 4; Fig. S1) (24). The periplasmic protease CtpA then processively degrades the remainder of the FhaB^CP^ prodomain to form a polypeptide called FHA′ (~263 kD) that lacks a functional PNT and hence ultimately exits the FhaC channel (25). Under certain conditions, such as prolonged growth *in vitro*, the surface-anchored autotransporter protease SphB1 is required for cleavage near the FHA′ C terminus, causing the immediate release of a ~ 250 kD polypeptide called FHA (Fig. S1). SphB1 is required to cleave FhaB precursors at two alternative sites just N-terminal to the FHA cleavage site to form FHA_1_ and FHA_2_, and this activity is enhanced in the absence of CtpA (i.e., when the prodomain cannot be fully degraded) (25). Although *sphB1* is co-regulated with *fhaB* in both *B. pertussis* and *B. bronchiseptica* (22, 26, 27) and SphB1-dependent cleavage of FhaB has been reported to be a critical maturation process (28), the biological significance of SphB1 cleavage of FhaB is unclear.

ACT is a large (1,706 aa) bifunctional protein. Its N-terminal adenylate cyclase domain (ACD) binds calmodulin and catalyzes the production of 3′−5′-cyclic AMP (cAMP) from ATP (29, 30). Its C-terminal RTX hemolysin domain contains (from N terminus to C terminus) a hydrophobic pore-forming subdomain, a segment that is palmitoylated on lysine residues by the *cyaC* gene product (31, 32), a calcium-binding RTX subdomain composed of 45 nonapeptide repeats, and a signal for export by the Type I secretion system that is formed by the products of the *cyaB*, *cyaD*, and *cyaE* genes (33, 34). A DDE motif within the nonapeptide repeats mediates the binding of ACT to the CD11b component of CR3 (CD11b/CD18, Mac1), a receptor present in macrophages, dendritic cells, and neutrophils (35, 36).

During culture in standard growth medium for bordetellae (Stainer Scholte broth), ACT, *via* its AC domain, binds FhaB on the surface of *B. pertussis* and *B. bronchiseptica*, and this binding prevents FhaB-FhaB interactions that are critical for biofilm formation (6). Hence, *Bordetella* species form robust biofilms when growing in BvgAS-intermediate (BvgAS^i^) mode during which they produce FhaB but not ACT (7, 37). BvgAS is a two-component regulatory system that activates the expression of all protein virulence factor-encoding genes. When it is partially active (BvgAS^i^ mode), it activates the expression of genes with high-affinity BvgA-binding sites at their promoters, such as *fhaB*, but not those with low-affinity BvgA-binding sites, such as *cyaA*. Strains lacking ACT also form biofilms under BvgAS^+^ mode conditions (6). Antibodies against the FhaB mature C-terminal domain block ACT from binding to FhaB (6), suggesting that the AC domain binds specifically to the FhaB mature C-terminal domain, but details about the ACT-FhaB interaction are lacking.

The terminally processed FHA protein was considered for many years to be the functional form of FhaB. However, we showed several years ago that *B. bronchiseptica* strains producing FhaB proteins lacking the extreme C terminus or the adjacent proline-rich region are defective for persistence in the lower respiratory tracts of mice, despite adhering to host cells *in vitro* as effectively as wild-type bacteria (11). Because the prodomain is degraded in the periplasm and does not function as a stand-alone polypeptide, these data indicate that full-length FhaB plays an important role in defense against phagocytic cells in the lower respiratory tract during infection. Given the importance of ACT in defense against phagocytic cells (9, 38–42), and the fact that it can bind to FhaB on the bacterial cell surface, we have speculated that FhaB may serve as a novel toxin delivery system that controls the delivery of ACT specifically to phagocytic cells (12, 24). According to this model, newly secreted ACT binds FhaB (i.e., in complex with FhaC) on the bacterial surface (Fig. 1 step 3). The binding of FhaB-associated ACT to CR3 on phagocytes generates a signal that is propagated across the bacterial outer membrane, resulting in a conformational change in the FhaB prodomain such that DegP cleaves off the FhaB extreme C terminus, and the new C terminus is then degraded by CtpA (Fig. 1 steps 4 and 5). We hypothesize that degradation of the prodomain allows the C terminus of FHA′ to slide through FhaC to the cell surface, permitting ACT to be released from FhaB and delivered to the phagocytic cell (Fig. 1 steps 6 and 7). Here, we examined specific aspects of this model. We determined that secreted ACT preferentially binds FhaB on the bacterial surface. We determined that ACT undergoes SphB1-dependent cleavage and we identified the dominant cleavage site, and we determined that *B. bronchiseptica* can deliver FhaB-bound ACT to phagocytic host cells.

## RESULTS

### FhaB efficiently retains newly secreted ACT on the *B. bronchiseptica* cell surface

ACT interacts with FhaB on the surface of both *B. bronchiseptica* and *B. pertussis* (6, 43). However, studies identifying these interactions did not examine the dynamics of ACT-FhaB binding. We hypothesize that ACT binds FhaB during, or immediately upon, secretion, before being released into culture supernatants. To test this hypothesis, we sought to track the amount and localization of newly secreted ACT in wild-type and ∆*fhaB* bacteria. We degraded surface-associated, external ACT with Proteinase K (Prot K), washed the bacteria to remove the enzyme and supernatant polypeptides, sub-cultured bacteria in fresh medium, and compared the amount of ACT on the bacterial surface, in culture supernatants, and whole cell lysates (WCL) *via* dot and western blot analyses.

**Fig 2:**
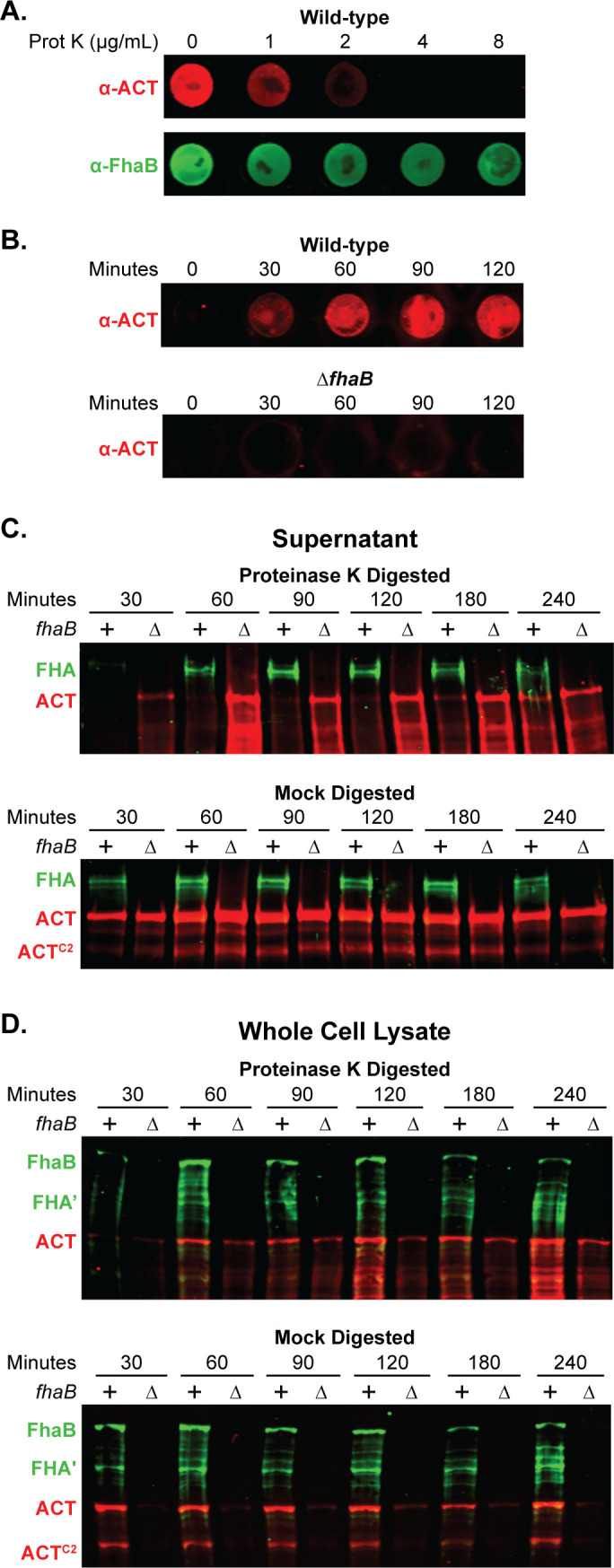
Newly secreted ACT binds FhaB on the cell surface prior to release. (**A)** To degrade surface-exposed proteins, wild-type bacteria were treated for 30 minutes with varying concentrations of Proteinase K (Prot K) and then washed. Surface ACT and FhaB were probed for *via* dot blot of intact cells using polyclonal α-ACT and α-FhaB antibodies. (B) Wild-type and Δ*fhaB* strains were treated with 4 µg/mL of Prot K for 30 minutes, washed, and cultured further in fresh media. Bacteria were sampled at the times listed to probe by dot blot for surface-associated ACT. (C and D) Wild-type and Δ*fhaB* strains were treated with 4 µg/mL of Prot K for 30 minutes (or mock digested with solution lacking Prot K), washed, and sub-cultured for the indicated times. Production, processing, and release of FhaB and ACT were examined by western blot analyses of filtered supernatants (**C**) and of whole cell lysates (WCL) (**D**), using polyclonal α-FhaB and monoclonal α-ACT antibodies that recognize the MCD and the AC domain C terminus (3D1), respectively. The images shown are representative of at least three biologically independent experiments.

To determine the appropriate concentration of Prot K for these experiments, we treated wild-type bacteria with 0–8 µg/mL Prot K for 30 minutes, washed the bacteria thoroughly, and performed dot blots on intact bacteria using FhaB- and ACT-specific antibodies. 4 µg/mL Prot K was sufficient to degrade ACT to levels undetectable by dot blot while leaving FhaB, which is intrinsically resistant to proteolysis by Prot K (22), still detectable (Fig. 2A). We used a 4 µg/mL concentration of Prot K for subsequent experiments.

We next determined the time required for newly secreted ACT to accumulate at the bacterial surface to levels detectable by dot blot following treatment with Prot K. Intact bacteria collected at 0, 30, 60, 90, and 120 minutes post-treatment were probed with ACT-specific polyclonal antibodies. ACT was detected on the surface of wild-type bacteria beginning at 30 minutes, and the amount increased for the following hour (Fig. 2B). By contrast, no ACT was detected on the surface of the ∆*fhaB* strain even after 120 minutes of post-treatment culturing, supporting previous findings that FhaB is required to retain ACT on the *B. bronchiseptica* surface (Fig. 2B). In culture supernatants of ∆*fhaB* bacteria (normalized to the OD_600_ of the culture), ACT was detectable by western blot at 30 minutes post-Prot K treatment and increased over time (Fig. 2C). By contrast, ACT was not appreciably detected in culture supernatants of wild-type bacteria until 180 minutes post-Prot K treatment and did not match levels present in supernatants of ∆*fhaB* bacteria until 240 minutes post-treatment (Fig. 2C). Without Prot K digestion (mock digested), amounts of ACT in culture supernatants of wild-type and ∆*fhaB* bacteria were equivalent at every time point.

In contrast to supernatants, cell-associated ACT was detected in the WCL of wild-type and ∆*fhaB* bacteria at 60 minutes post-treatment with Prot K, and all timepoints after that (Fig. 2D). In all cases, the amount of ACT present in the WCL of the ∆*fhaB* strain was less than that present in WCL of wild-type bacteria. The ACT detected in WCL of the ∆*fhaB* mutant was likely newly synthesized, cytoplasmic ACT as it was not detected by dot blot (Fig. 2B). Mock-treated wild-type and ∆*fhaB* bacteria secreted similar amounts of ACT at all times tested (Fig. 2C). Collectively, these data suggest that secreted ACT efficiently binds FhaB on the cell surface until ACT has bound all available FhaB, at which point excess toxin is released directly or in complex with FhaB into the medium.

### ACT inhibits FhaB processing

**Fig 3:**
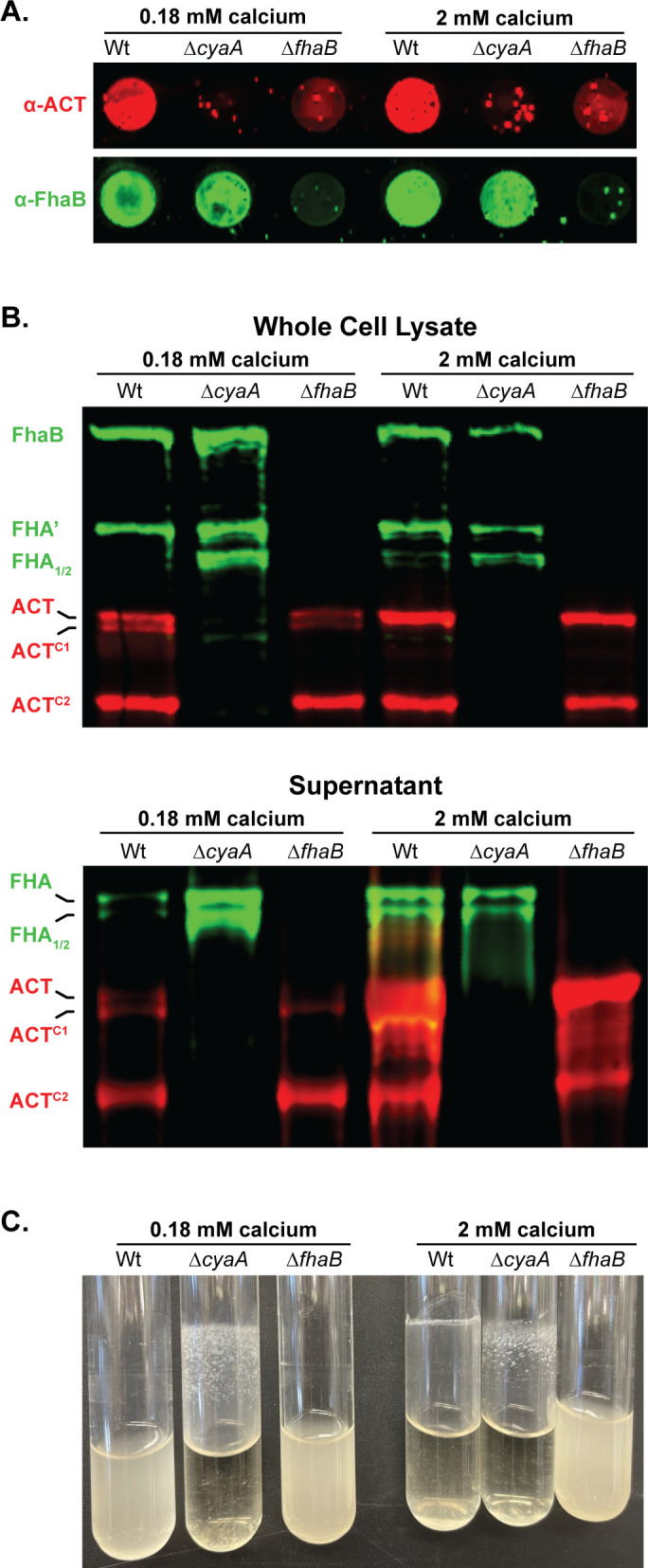
Calcium does not prevent the association of FhaB and ACT on the bacterial surface of *B. bronchiseptica*. (**A)** Dot blots of intact bacteria cultured for 20 hours in standard Stainer Scholte (SS) broth, which has 0.18 mM CaCl_2_, or SS supplemented with 1.8 mM additional CaCl_2_ (bringing the total to 2 mM CaCl_2_). Blots were probed with polyclonal α-ACT and α-FhaB antibodies. (B) Western blot analyses of whole cell lysates and culture supernatants grown in conditions described in (A). Blots were probed with polyclonal α-FhaB and 3D1 monoclonal α-ACT antibodies. Full-length and multiple cleaved forms of ACT and FhaB are indicated. (C) Culture tubes of wild-type, Δ*cyaA*, and Δ*fhaB* bacteria were cultured for 20 hours in standard SS broth or SS supplemented with additional CaCl_2_. The adherent band occurs at the liquid-air-glass interface while rotating tilted. The images shown are representative of at least three biologically independent experiments.

To determine whether the presence of ACT affects the processing of FhaB, we compared FhaB and ACT profiles in wild-type and ACT-deficient strains by western blot and dot blot analyses using anti-FhaB (green in Fig. 3) and anti-ACT (red in Fig. 3) antibodies. Because it has been reported that Ca^2+^ concentration in the medium influences ACT localization (44), we grew bacteria in either standard SS broth (15), which contains 0.18 mM Ca^2+^, or in SS broth containing 2.0 mM Ca^2+^. ACT was detected on the surface of wild-type, but not ∆*fhaB* or ∆*cyaA*, bacteria grown in both media (Fig. 3A), indicating that ACT remains associated with the bacterial surface in a FhaB-dependent manner regardless of Ca^2+^ concentration.

**Fig 4:**
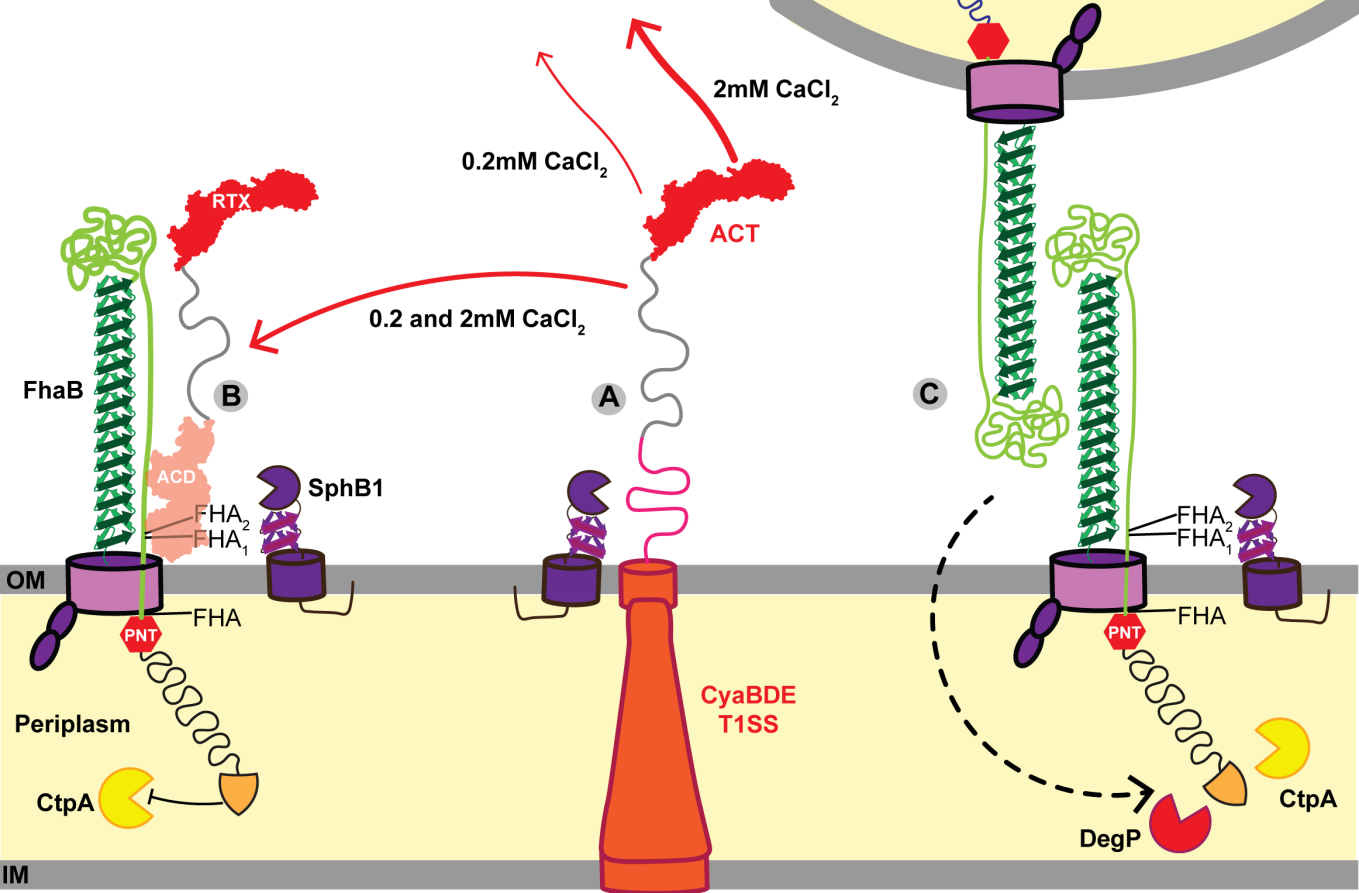
Model of the interactions between FhaB, ACT, and SphB1 on the surface of *Bordetella bronchiseptica*. (**A)** The rate of ACT secretion through the CyaBDE Type I secretion system is higher when the bacteria are grown in SS broth with 2 mM CaCl_2_ than in broth with 0.2 mM CaCl_2_. In the absence of FhaB, ACT undergoes SphB1-dependent cleavage at multiple sites within the ACD and TM domains on the cell surface, presumably as ACT exits the T1SS channel. (B) The N-terminal ACD of ACT binds the extracellular MCD of FhaB on the surface of wild-type bacteria blocking SphB1-dependent cleavage of FhaB at the FHA_1/2_ cleavage sites regardless of CaCl_2_ concentration. (C) In the absence of ACT, FhaB molecules interact with abiotic surfaces and FhaB on adjacent bacteria, resulting in biofilm formation and bacterial aggregation. ACT-deficient strains exhibit increased SphB1-dependent cleavage of FhaB to form FHA_1/2_ as well as increased DegP and CtpA processing of the FhaB prodomain that may be triggered by FhaB-FhaB or FhaB-surface interactions.

WCL of wild-type bacteria grown in SS typically contains full-length FhaB and FHA′, the FhaB polypeptide remaining after DegP- and CtpA-mediated degradation of the prodomain (24, 25) (Fig. 3B, first lane (0.18 mM calcium), and see Fig. 1; Fig. S1). In the absence of ACT (∆*cyaA*), FHA_1_ and FHA_2_, products of SphB1-dependent cleavage, were also present (Fig. 3B, second lane). Culture supernatants of wild-type bacteria grown in SS typically contain FHA, FHA_1_, and FHA_2_, and all of these polypeptides were more abundant in culture supernatants of the ∆*cyaA* strain compared to wild-type bacteria (Fig. 3B, 0.18 mM calcium). ACT-dependent differences in FhaB processing and secretion were not apparent in bacteria grown in SS containing 2.0 mM Ca^2+^ (Fig. 3B, right lanes). Increased production of FHA, FHA_1_, and FHA_2_ in the absence of ACT indicates that the presence of ACT inhibits SphB1-dependent cleavage of FhaB and the concomitant release of the resulting polypeptides, which we hypothesize is due to steric hindrance by FhaB-bound ACT (depicted in Fig. 4B), especially prominent when the bacteria are grown in standard SS medium. SphB1-dependent cleavage can convert full-length FhaB to FHA_1_ and FHA_2_, even when the prodomain is intact because the sites in FhaB that are cleaved to generate FHA_1_ and FHA_2_ are present on the bacterial surface (see Fig. 4B and C). However, the site in FhaB that is cleaved to generate FHA is not present on the cell surface until the prodomain N terminus (PNT) has been degraded, allowing the C-terminus of FHA′ to slide through FhaC to the outside of the cell (25). The increased abundance of FHA in culture supernatants from ∆*cyaA* cultures indicates that there is increased DegP- and CtpA-dependent degradation of the prodomain in the absence of ACT (Fig. 3B). These data suggest that ACT plays a role in the signaling event that initiates prodomain degradation (Fig. 4). That is, ACT binding may stabilize FhaB in a conformation that reduces regulated degradation of the prodomain by DegP and CtpA proteases in the periplasm.

We observed that ACT is also cleaved by an as yet unreported protease into a smaller polypeptide which we have labeled ACT^C2^ (Fig. 2 and 3). We address the formation of the ACT^C2^ product in the subsequent results section. ACT processing and release were not affected by the presence or absence of FhaB, but, consistent with Bumba et al. (44), there was substantially more ACT in culture supernatants of bacteria grown in 2.0 mM Ca^2+^ (despite there being similar ACT levels in WCL), compared with bacteria grown in SS containing 0.18 mM Ca^2+^ (Fig. 3B and C). This result is consistent with experiments done with *B. pertussis* that showed that 2.0 mM Ca^2+^ facilitates the folding of the RTX repeats in ACT, which increases secretion efficiency (44). Together, these data indicate that although increased Ca^2+^ may increase ACT folding and secretion, it does not prevent ACT from binding to FhaB.

Wild-type and ∆*fhaB B. bronchiseptica* grow planktonically in standard SS medium, while the ∆*cyaA* strain grows as a biofilm adherent to the walls of the culture tube (Fig. 3C). These data are consistent with previously published reports showing that biofilm formation in *B. pertussis* and *B. bronchiseptica* requires FhaB-FhaB interactions that can be inhibited by FhaB-ACT interactions (5–7). When grown in SS containing 2.0 mM Ca^2+^, wild-type bacteria also aggregated and formed a ring in the culture tubes, but in a way that was visibly different from the biofilm formed by the ∆*cyaA* strain (Fig. 3C). These data indicate that the ACT bound to FhaB in bacteria grown in 2.0 mM Ca^2+^ [which can be detected by dot blot (Fig. 3A)] is insufficient (possibly due to being in a different conformation and/or there being fewer ACT-ACT interactions) to completely block the FhaB-FhaB interactions required for biofilm formation.

### ACT is cleaved in an SphB1-dependent manner

**Fig 5:**
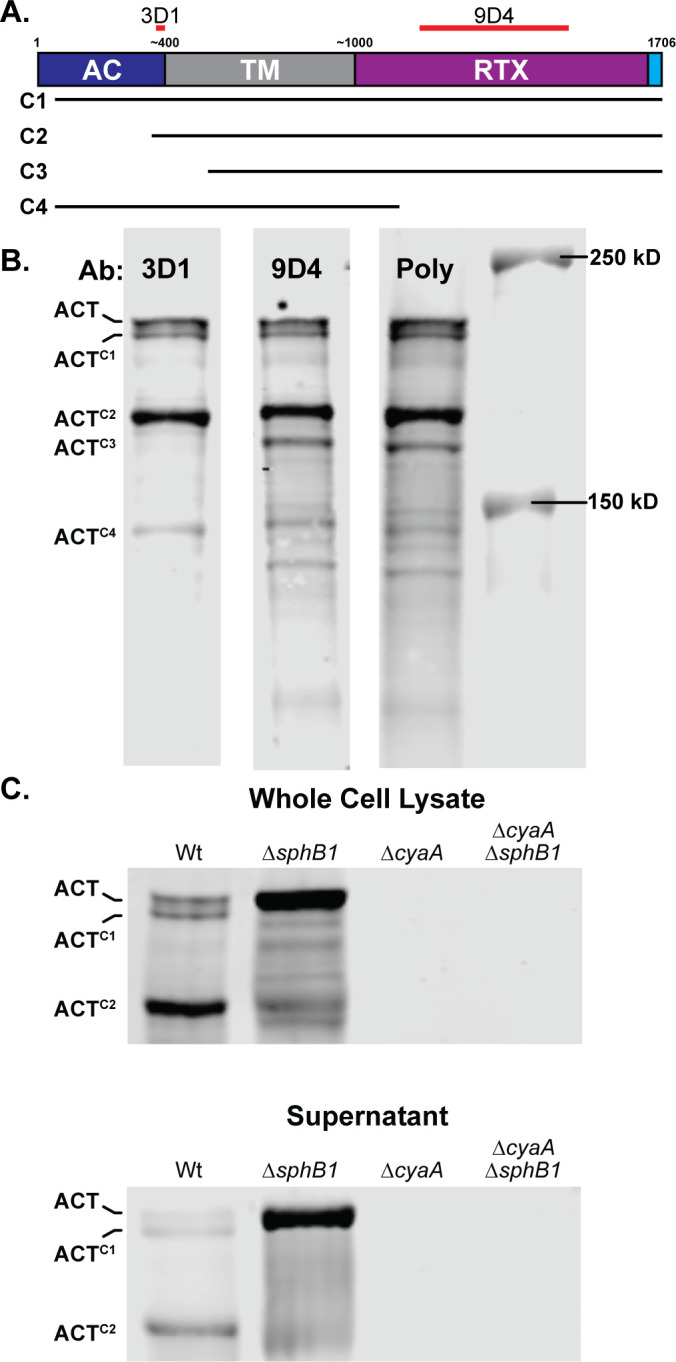
ACT is cleaved in an SphB1-dependent manner. (**A)** Schematic of ACT, showing adenylate cyclase domain (AC, blue), transmembrane domain (TM, grey), repeats-in-toxin domain (RTX), and C-terminal Type I signal sequence (cyan). Estimated and actual amino acid markers are shown at the top. Regions used to generate monoclonal antibodies 3D1 and 9D4 are indicated with red bars overlapping residues 373–399 and 1,156–1,489, respectively. Predicted portions of ACT remaining in the ACT^C1^, ACT^C2^, ACT^C3^, and ACT^C4^ polypeptides are indicated with lines underneath the diagram, although the exact sizes cannot be determined by examining these blots. (B) Western blots of whole cell lysates from wild-type bacteria were probed with monoclonal antibodies 3D1 or 9D4 or with polyclonal antibodies generated using whole ACT protein (Poly). (C) Western blot analyses of *B. bronchiseptica* strains lacking SphB1 and/or ACT. Blots are focused on three forms of ACT in both whole cell lysates and culture supernatants: uncleaved ACT and two cleaved forms ACT^C1^ and ACT^C2^. Blots were probed with 3D1 α-AC domain monoclonal antibody. The images shown are representative of at least three biologically independent experiments.

Although the primary amino acid sequence of ACT predicts it to be a 177 kD polypeptide, ACT typically runs with the mobility of a larger ~200 kD protein by SDS-PAGE, due, likely, to folding of the RTX repeats. A substantial amount of a smaller polypeptide running with an apparent MW of ~175 kD (ACT^C2^) was also detected with the ACT-specific 3D1 monoclonal antibody in the western blots shown in Fig. 2 and 3 (see Fig. 5B for MW markers). To investigate the nature of those polypeptides, we probed WCL of wild-type *B. bronchiseptica* with a monoclonal antibody that recognizes an epitope near the C terminus of the AC domain (3D1) (45), a monoclonal antibody that recognizes an epitope within the RTX domain (9D4) (45), and a polyclonal antibody raised against the entire ACT protein (Poly) (46) (Fig. 5B). The most prominent polypeptides, ACT, ACT^C1^, and ACT^C2^, as well as a less abundant polypeptide running at about 140 kD (ACT^C4^), were recognized by all three antibodies (Fig. 5B). A polypeptide running at ~170 kD (ACT^C3^) was recognized by the 9D4 monoclonal antibody and the polyclonal antibody, but not 3D1 (Fig. 5B), indicating ACT^C3^ contained the C terminus of ACT and not the AC domain. To determine whether cleavage of ACT is dependent on SphB1, we compared ACT profiles in WCLs and supernatants from wild-type and ∆*sphB1* cultures using samples from the ∆*cyaA* and ∆*cyaA* ∆*sphB1* strains as controls. Western blot analysis with the 3D1 antibody showed that ACT was the predominant or only form of ACT in WCL and supernatants of ∆*sphB1* bacteria (Fig. 5C), demonstrating that cleavage of ACT is indeed SphB1-dependent.

There was more full-length ACT relative to ACT^C2^ in supernatants collected from *B. bronchiseptica* grown with 2 mM CaCl_2_ than in standard SS (Fig. 3B). To determine whether calcium reduces the level or alters the localization of SphB1, we generated a strain of *B. bronchiseptica* producing SphB1 with an HA-epitope inserted after proline 58 and examined SphB1-HA protein levels in WCLs and supernatants collected from bacteria grown in either standard SS or SS with 2 mM CaCl_2_. Similar amounts of an approximately 70 kD protein were observed in HA-specific western blots of WCLs and supernatant fractions from bacteria grown in both low and high calcium (Fig. S2). The size of the *B. bronchiseptica* SphB1^NT-HA^ polypeptide is similar to mature *B. pertussis* SphB1 polypeptide, which has been reported to migrate as a ~75 kD polypeptide after auto-catalytic cleavage during secretion (28). These data indicate that the amount and localization of SphB1 are not affected by the calcium concentration of the media used in this study.

### Identification of the SphB1-dependent cleavage site within the ACT AC domain

**Fig 6:**
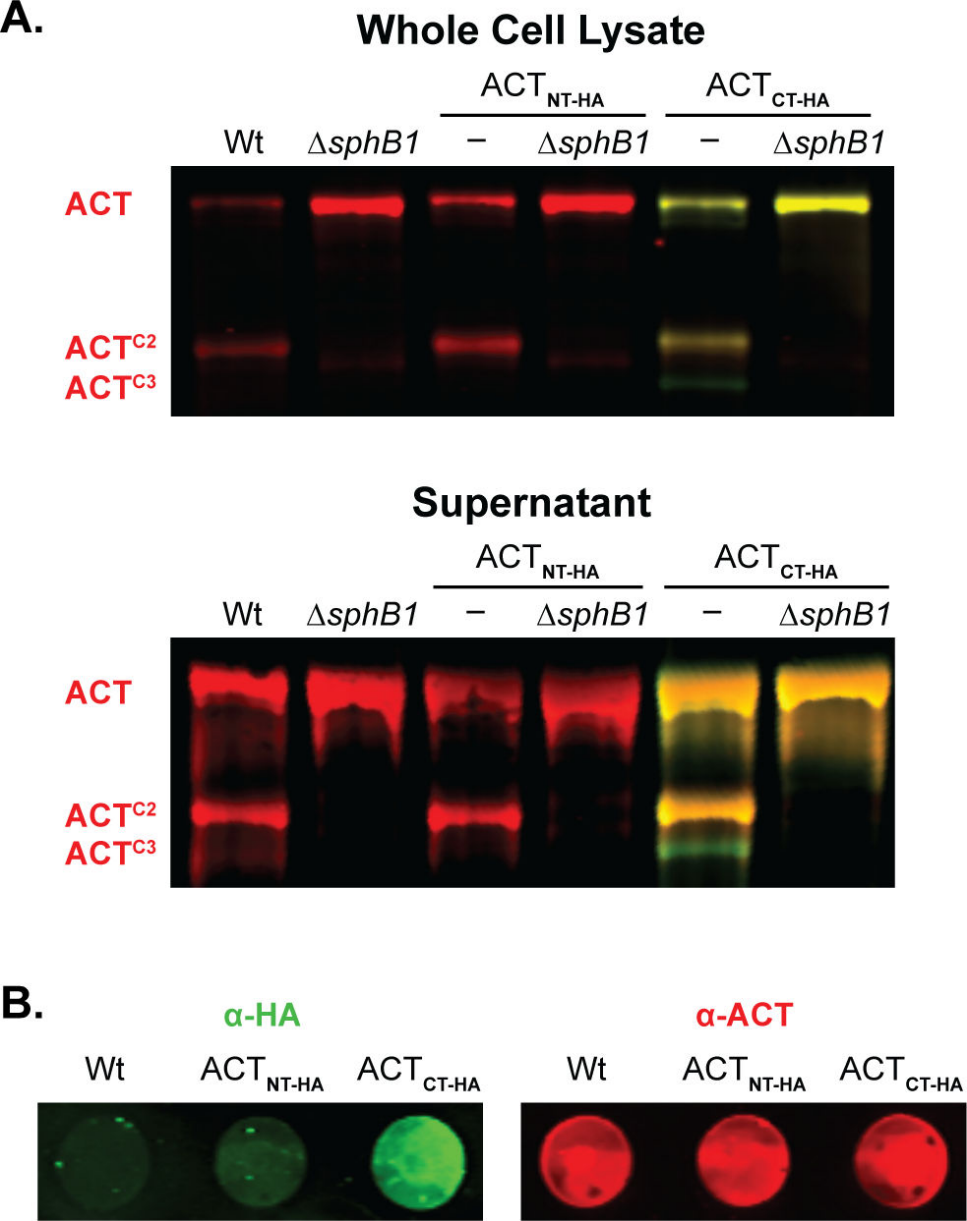
SphB1-dependent cleavage of ACT occurs at the N-terminus. (**A)** Western blot analyses of HA-tagged ACT, with the HA tag at either the N or C terminus (ACT^NT-HA^ and ACT^CT-HA^, respectively). Red bands indicate ACT polypeptides that possess residues 373–399 (the AC domain C terminus recognized by monoclonal antibody 3D1, see Fig. 4). Yellow bands indicate polypeptides that possess ACT residues 373–399 and also an HA tag (labeled with HA-specific polyclonal rabbit antibodies). Green bands indicate polypeptides that lack residues 373–399 (the 3D1 epitope) but possess a C-terminal HA-tag. (B) Dot blots of intact wild-type bacteria and strains that produce HA-tagged versions of ACT. Bacteria were separately probed for HA (green) or ACT (red) using polyclonal rabbit antibodies. The images shown represent at least three biologically independent experiments.

To characterize the SphB1-dependent ACT cleavage products further, we constructed strains producing ACT with an HA epitope at either the N or C terminus and examined protein profiles *via* western blotting with the 3D1 ACT antibody (red in Fig. 6) and polyclonal rabbit anti-HA antibodies (green in Fig. 6). The N-terminal HA epitope was barely detected in the intact ACT polypeptide (and not detected in the smaller ACT polypeptides) (Fig. 6; Fig. S3), indicating that the N-terminal HA epitope was proteolytically removed from a majority of the ACT polypeptides. The C-terminal HA epitope was present on the full-length ACT_CT-HA_ protein, ACT^C1^, ACT^C2^, and ACT^C3^, indicating that SphB1-dependent cleavage to produce ACT^C1^ and ACT^C2^ occurs within the AC domain (Fig. 6A). Consistent with the western blot results, the C-terminal, but not N-terminal, HA epitope was detected on the cell surface by dot blot (Fig. 6B). These data indicate that SphB1-dependent cleavage of ACT occurs at three or more distinct sites within ACT.

**Fig 7:**
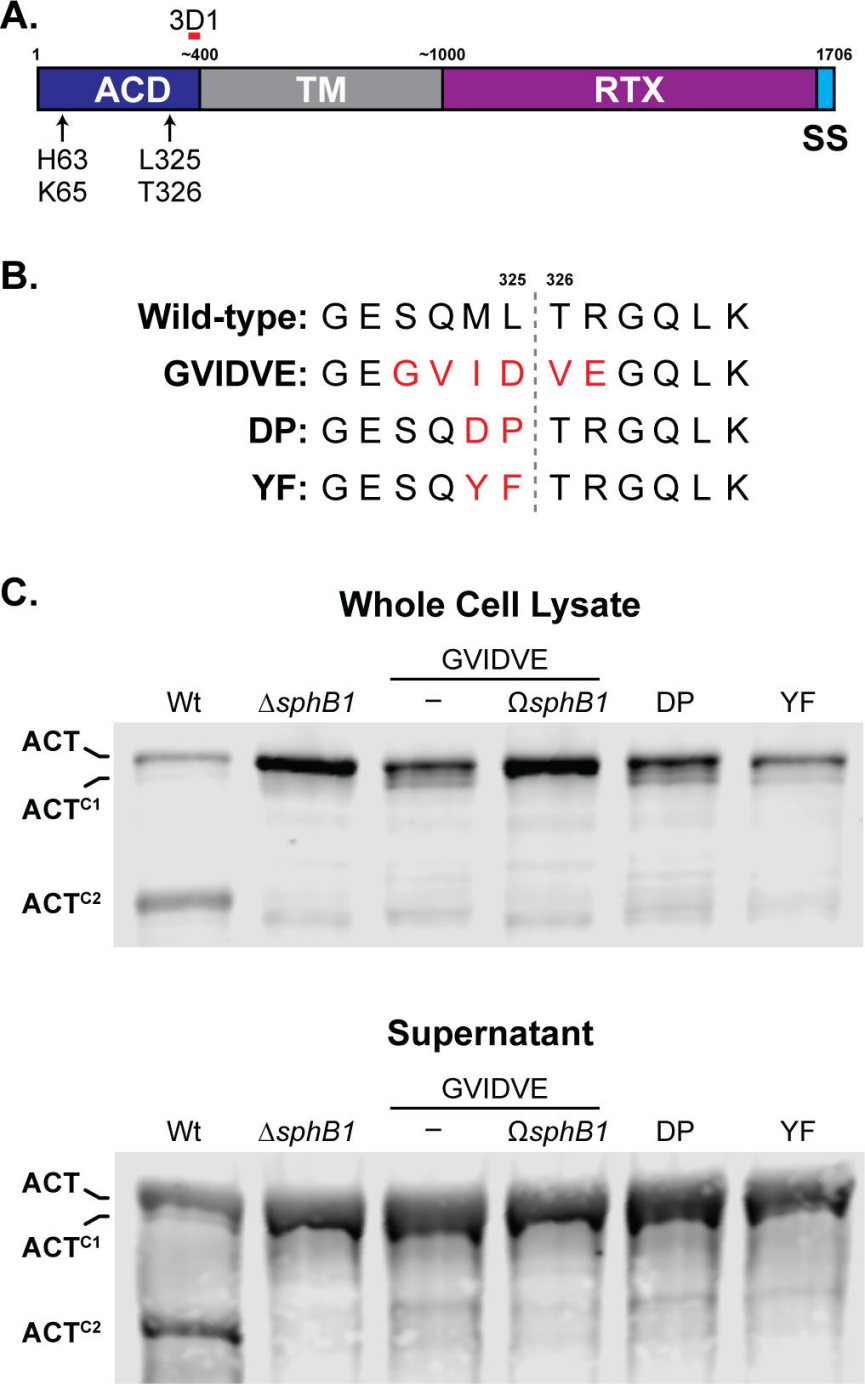
Identification of the SphB1-dependent cleavage site on ACT. (**A)** Schematic of ACT, showing adenylate cyclase domain (ACD), transmembrane domain (TM), repeats-in-toxin domain (RTX), and C-terminal signal sequence (SS). Estimated and actual amino acid markers are shown at the top. AC catalytic residues histidine 63 and lysine 65 are indicated, as well as the predicted SphB1-dependent cleavage site between leucine 325 and threonine 326 that yields ACT^C2^. The monoclonal antibody 3D1 recognition region is indicated by a short red bar overlapping residues 373–399. (B) Sequence of the predicted SphB1-dependent cleavage site within the AC domain compared between wild-type ACT and three amino acid substitution mutants. Substitutions are highlighted in red, and the cleavage location between L325 and T326 is marked by a gray dotted line. (C) Western blot analyses of whole cell lysates and culture supernatants of strains with ACT modifications as shown in (B). Also included are wild-type, Δ*sphB1*, and GVIDVE with an insertional disruption of *sphB1* (GVIDVE Ω*sphB1*). Blots were probed with 3D1 antibody, and the three primary ACT forms were marked at left. The images shown are representative of at least three biologically independent experiments.

ACT^C2^ was the most abundant ACT polypeptide in both supernatants and WCLs of overnight cultures of wild-type bacteria. We used Edman sequencing to determine that the ACT^C2^ N terminus is TRLGQLKEY, which matches the aa sequence spanning T326-Y334 within the ACT N-terminal adenylate cyclase domain (ACD). This result indicates that the SphB1-dependent cleavage event that generates ACT^C2^ occurs between L325 and T326 (dashed line in Fig. 7B) and that ACT^C2^ contains the anti-3D1 epitope. We used allelic exchange to construct three strains producing ACT with aa substitutions in and around the identified cleavage site (changed residues shown in red in Fig. 7B). The SphB1-dependent ACT^C2^ polypeptide was not detected in WCLs or supernatants from *B. bronchiseptica* producing the altered forms of ACT (Fig. 7C), indicating that the amino acid substitutions abrogated SphB1-dependent cleavage of ACT at this site. As expected, ACT^C1^ was present in WCLs of the cleavage site mutants and was absent in the GVIDVE mutant in which *sphB1* was disrupted (*ΩsphB1,* Fig. 7C). Dot blots of these strains using ACT- and FhaB-specific antibodies showed similar amounts of ACT^GVIDVE^, ACT^M324D/L325P^(DP), and ACT^M324Y/L325F^ (YF) on the surface of the mutant strains as ACT on wild-type bacteria but not the ACT^GVIDVE^ ∆*fhaB* mutant. These data indicate that FhaB is required to retain the altered ACT proteins on the bacterial surface and that the aa substitutions in ACT did not disrupt the association between the ACD and FhaB (Fig. S4).

### SphB1-dependent cleavage of ACT does not require surface retention of ACT by FhaB

**Fig 8:**
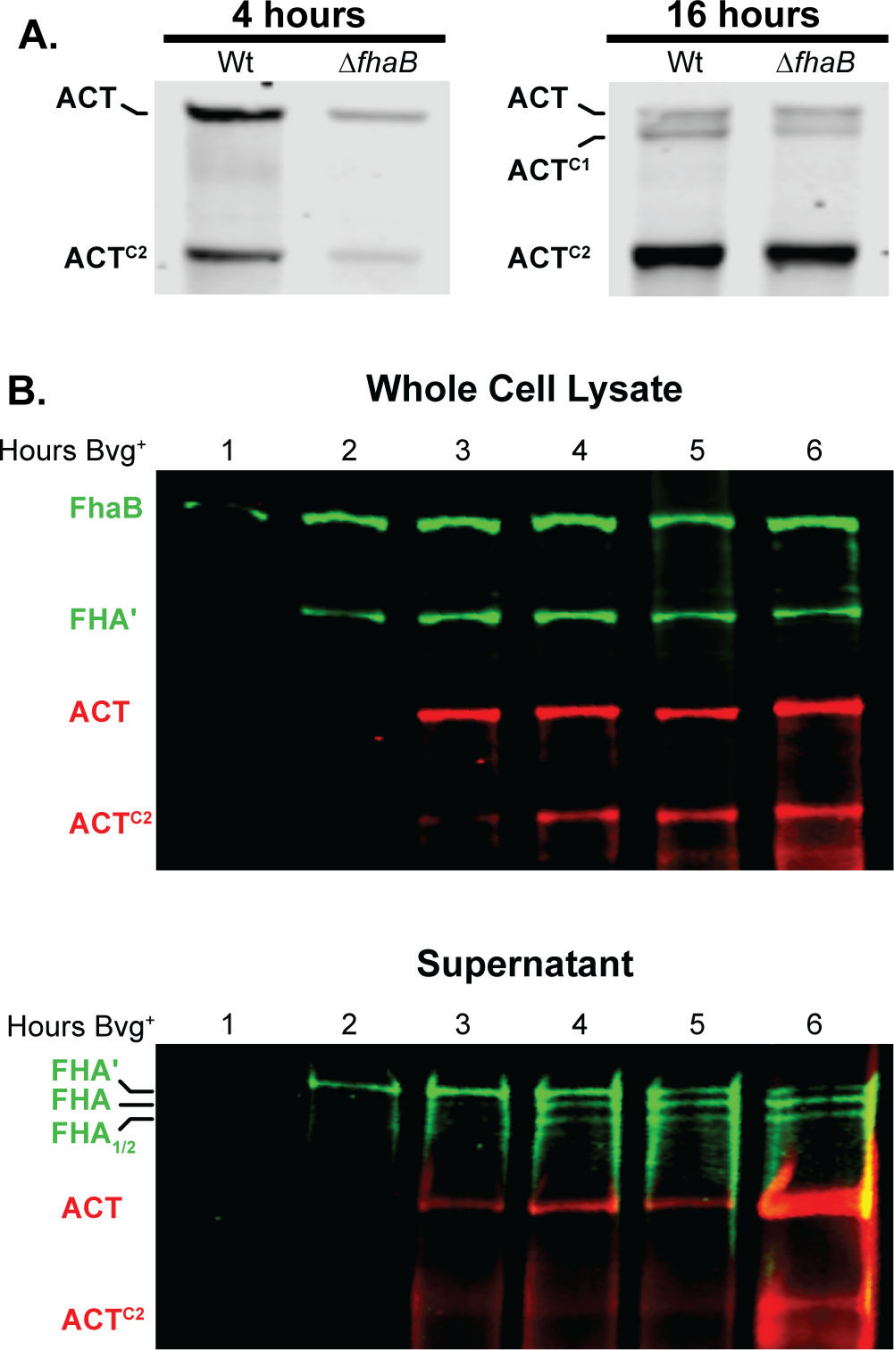
Production and degradation of FhaB and ACT over time. (**A**) ACT degradation in whole cell lysates of wild-type bacteria that were grown for 4 hours or 16 hours in BvgAS-inducing media. ACT was probed *via* western blot with the 3D1 α-ACT monoclonal antibody. (B) Production, degradation, and release of FhaB and ACT through 6 hours post-induction. Wild-type bacteria were subcultured in BvgAS-inducing media (SS media) following overnight growth in non-inducing media (SS media with 50 mM MgSO_4_), and whole cell lysates and culture supernatants were probed *via* western blot analyses with α-FhaB polyclonal and the 3D1 α-ACT monoclonal antibodies. Full-length proteins and the major degraded polypeptides are indicated. The images shown are representative of at least three biologically independent experiments.

To determine whether SphB1-dependent cleavage of ACT requires ACT’s interaction with FhaB, we compared ACT protein profiles in wild-type and ∆*fhaB* strains. For these experiments, we grew bacteria in a medium containing 50 mM MgSO_4_, a condition in which BvgAS, the two-component regulatory system that activates expression of *fhaB* and *cyaA,* is inactive, then switched the culture to BvgAS-activating conditions and collected samples at 4 hours and 16 hours post-shift. At 4 hours post-shift, ACT and ACT^C2^ were present in the WCL of wild-type and ∆*fhaB* strains (Fig. 8A), indicating that SphB1-dependent cleavage to form ACT^C2^ occurs even in the absence of FhaB. At 16 hours post-shift, ACT^C1^ was also present, suggesting cleavage to form ACT^C1^ also occurs in the absence of FhaB, but is less efficient than cleavage to form ACT^C2^. These data, as well as those shown in Fig. 3, indicate that SphB1-dependent cleavage of ACT does not require retention of ACT on the surface by FhaB.

To investigate the maturation of FhaB and ACT in more detail, we monitored FhaB and ACT protein profiles every hour for the first 6 hours post-shift from BvgAS-inactivating to BvgAS-activating conditions. In WCL, only full-length FhaB was detected at 1 hour post-shift (Fig. 8B). FHA′, resulting from degradation of the prodomain by DegP and CtpA, was detectable at 2 hours post-shift, and ACT was first detected at 3 hours post-shift. ACT^C2^ was detected at 4 hours post-shift. In culture supernatants, SphB1-dependent FHA and FHA_1/2_ were detected at about 3–4 hours post-shift, while ACT^C2^ was not detected until about 6 hours post-shift. These data indicate a sequence of events in which FhaB is produced, then processed by DegP and CtpA and then SphB1, and then ACT is produced and then cleaved by SphB1 *in vitro*.

### SphB1-dependent cleavage of ACT inactivates ACT

**Fig 9:**
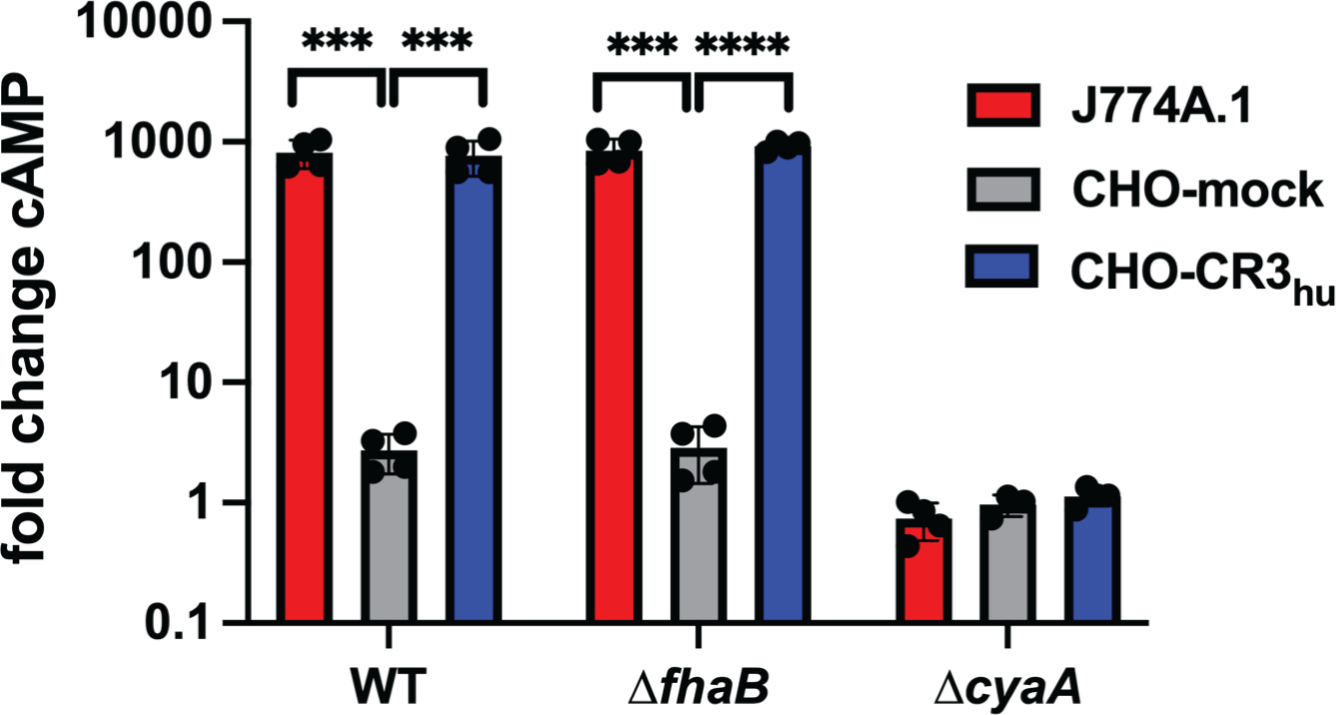
*B. bronchiseptica* intoxicates CR3-positive cells with ACT. J774A.1 macrophage cells (J774) and CHO-K1 Chinese hamster ovary cells containing integrated empty vector (CHO-mock) or vector with DNA that encodes the human complement receptor 3 (CHO-CR3_hu_) were infected with wild-type, ∆*fhaB,* or ∆*cyaA* bacteria for 30 minutes at an MOI of 100. cAMP amounts were determined by competitive ELISA and normalized to the fold change in cAMP by dividing the pmol cAMP/mL in infected cells by the pmol cAMP/mL in uninfected controls. The data shown are from four independent experiments and were analyzed using unpaired two-tailed *t*-tests. ***P < 0.001, ****P < 0.0001.

To investigate intoxication of host cells by *B. bronchiseptica*, we infected J774A.1 murine macrophage-like (CR3^+^) cells, Chinese hamster ovary cells carrying an empty vector (CHO-mock, CR3^–^), and CHO cells carrying the vector encoding human CD18 and CD11b (CHO-CR3_hu_^+^ (35)) with wild-type, ∆*cyaA*, and ∆*fhaB B. bronchiseptica* strains at a multiplicity of infection (MOI) of 100 and measured 3′−5′-cyclic AMP (cAMP) levels using a competitive ELISA. As has been shown for *B. pertussis* (35, 36)*,* wild-type *B. bronchiseptica* efficiently intoxicated CR3^+^ cells (J774A.1 and CHO-CR3_hu_+), but even at the high MOI of 100, it did not intoxicate CR3^–^ (CHO-mock) cells (Fig. 9), indicating that the CR3 receptor greatly enhances entry of ACT into host cells (35, 36). No increase in cAMP occurred in any of the cell types infected with the ∆*cyaA* strain. The lack of difference in cAMP induced by the wild-type and ∆*fhaB* strains may be due to the high MOI used and the fact that the bacteria were spun onto host cells to facilitate the interaction of the ∆*fhaB* strain with the host cells.

**Fig 10:**
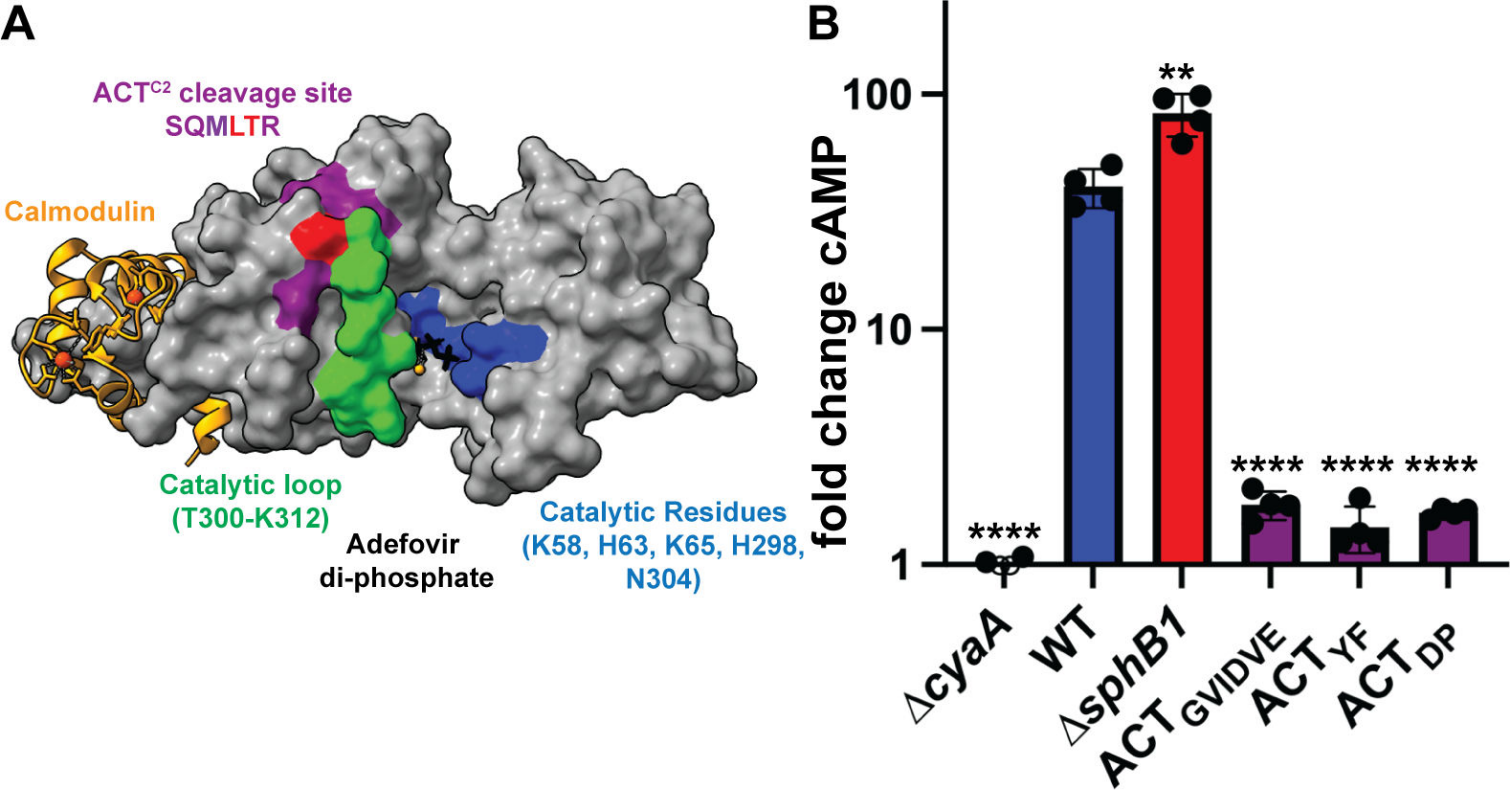
*B. bronchiseptica*-producing ACT with altered cleavage sites are defective for intoxication of macrophage-like cells *in vitro*. (**A**) The crystal structure of the *B. pertussis* ACT ACD (residues 7–364) in complex with Calmodlin-Ca^2+^ (orange) and the Adefovir di-phosphate nucleotide substrate analog (black) in the active site PDB:1ZOT (47). The residues flanking the site that is cleaved to form ACT^C2^ are shown in red (L325-T326), the residues proximal to the cleavage site that were mutated from SQMLTR to GVIDVE are shown in purple, residues required for catalytic activity are shown in blue, and the T300-K312 loop is shown in green. The cleavage site (red) is adjacent to the catalytic loop (green) in the folded protein. (B) J774A.1 cells were infected at an MOI of 10 with wild-type (WT), Δ*cyaA*, Δ*sphB1*, or the strains producing ACT with altered SphB1-dependent cleavage sites: ACT_GVIDVE_ (SQMLTR_322-328_GVIDVE), ACT_DP_ (ML_324-325_DP), ACT_YF_ (ML_324-325_YF). After 30 minutes of infection, cAMP amounts within the mammalian cells were determined by competitive ELISA and normalized by dividing the pmol cAMP/mL in infected cells by the pmol cAMP/mL in uninfected controls (fold change cAMP). The data shown are from four independent experiments and the results from each mutant strain were individually compared to the wild-type using unpaired two-tailed *t*-tests. **P < 0.01, ****P < 0.0001.

We mapped the location of the SQMLTR residues, where SphB1-dependent cleavage of ACT occurs, to a crystal structure of the *B. pertussis* ACD, which is identical to the *B. bronchiseptica* ACD at the aa level (Fig. S5), in complex with calmodulin and the nucleotide Adefovir diphosphate, within the ACT active site (PDB:1ZOT; (47)). The L325-T326 residues flanking the cleavage site (red, Fig. 10A) are proximally located to a loop (T300-K312, green) that is required to position N304 within the AC active site where it contacts the substrate. ACT catalytic activity is stimulated upon binding Calmodulin-Ca^2+^ within the cytoplasm of eukaryotic cells (29, 48, 49). Based on the crystal structure, the cleavage between aas L325-T326 to generate ACT^C2^ (Fig. 7) would separate the region of the ACD that binds calmodulin from the enzymatic active site, rendering ACT^C2^ catalytically inactive (Fig. 10A). To test this prediction, we compared the ability of wild-type and ∆*sphB1* mutant bacteria to intoxicate J774A.1 cells with ACT. We infected the mammalian cells at an MOI of 10 and measured cAMP levels as described above. The ∆*sphB1* strain, which contains full-length ACT in WCLs, on the bacterial surface, and in culture supernatants (see Fig. 5C and 6B), induced greater amounts of cAMP in J774A.1 cells than wild-type bacteria, which predominately have ACT^C2^ (Fig. 10B). This result is consistent with SphB1-dependent cleavage of ACT disrupting catalytic activity of the toxin. The location of SQMLTR residues within the folded ACT protein suggests that altering these residues could disrupt the AC activity of the modified proteins (Fig. 10B). To test this hypothesis, we examined the ability of bacterial strains producing ACT with aa substitutions in and around the ACT^C2^ cleavage site (Fig. 7) to intoxicate J774A.1 cells. All of these strains were defective for intoxication (Fig. 10B), indicating that these aa substitutions disrupted either toxin delivery or catalytic activity of ACT. The residues in ACT required to interact with CR3 are located distal to the ACT^C2^ cleavage site within the RTX domain (aa1193-1195 (35)), suggesting that in addition to preventing SphB1-dependent cleavage the aa substitutions made within the ACD disrupted catalytic activity.

### *B. bronchiseptica* delivers FhaB-bound-ACT to CR3^+^ mammalian cells

**Fig 11:**
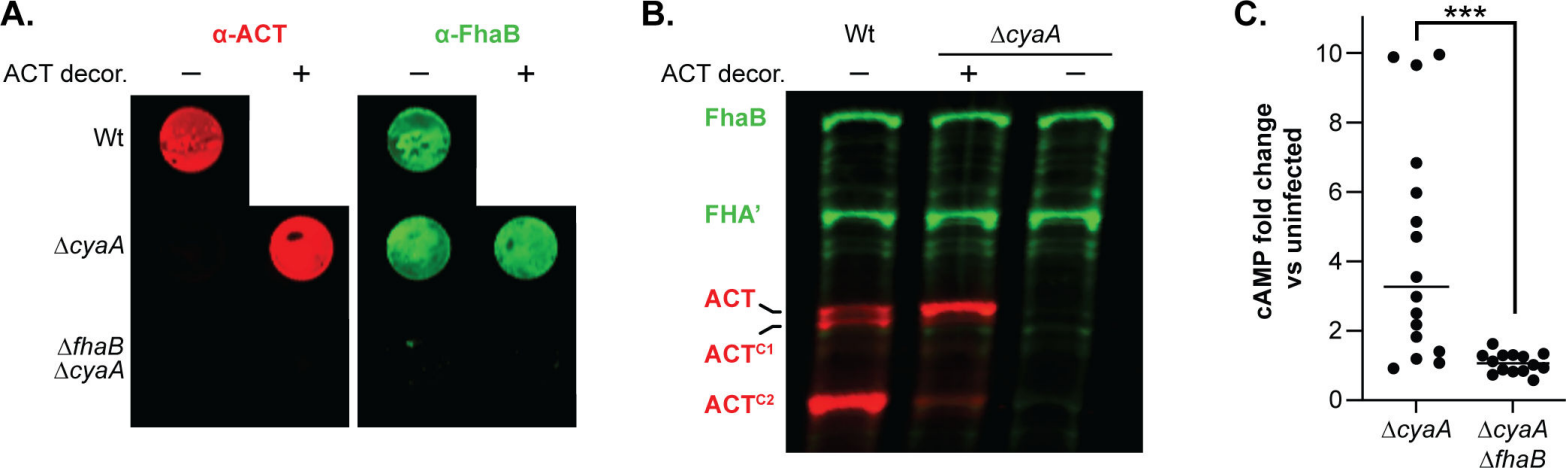
ACT bound to FhaB on the bacterial surface can be delivered to J774A.1 macrophage-like cells. (**A**) The surfaces of ACT-null bacteria (Δ*cyaA* and Δ*cyaA* Δ*fhaB* strains) were decorated with ACT released from Δ*fhaB* Δ*sphB1* bacteria by suspending the ACT-null bacteria in sterile-filtered, ACT-containing culture supernatant and then washing in PBS to remove non-adhered ACT. External, surface-bound ACT and FhaB were examined *via* dot blot of intact decorated and undecorated cells, probing with polyclonal α-ACT and α-FhaB antibodies. These images are representations of at least five independent experiments. (B) Western blot of wild-type, decorated Δ*cyaA*, and undecorated Δ*cyaA* bacteria. FhaB, ACT, and their cleaved forms were detected using polyclonal α-FhaB MCD and monoclonal 3D1 α-ACT antibodies. These images are representative of at least five independent experiments. (C) J774.1 macrophages were infected with decorated bacteria for 15 minutes at an MOI of 100. Levels of cAMP were determined by competitive ELISA and shown as fold change vs uninfected macrophages. The data shown are from 14 independent experiments and were analyzed by an unpaired, two-tailed *t*-test, ***P < 0.001.

It has been reported that FhaB-bound ACT is not delivered to host cells, that is, FhaB merely mediates adherence and it is newly secreted ACT that intoxicates the cell (50). To directly test the hypothesis that FhaB-bound ACT can be delivered to host cells, we incubated ∆*cyaA* strains with supernatants collected from cultures of the ∆*fhaB* ∆*sphB1* strain, which secretes large amounts of full-length, catalytically active, extracellular ACT so that the only ACT present on the bacteria would be that bound to FhaB on the cell surface (Fig. 11). We confirmed ACT “decoration” of the ∆*cyaA* strain by examining ACT levels in WCLs and on the bacterial surface after thoroughly washing the decorated bacteria (Fig. 11A). The ∆*cyaA* ∆*fhaB* strain did not retain ACT, indicating that FhaB is required for ACT decoration (Fig. 11A). cAMP levels in J774A.1 cells infected with the ACT-decorated ∆*cyaA* strain were significantly greater than J774A.1 cells infected with the ∆*cyaA* ∆*fhaB* strain (Fig. 11C). These data indicate that ACT bound to FhaB can be delivered to CR3^+^ host cells *in vitro*.

## DISCUSSION

Demonstration that ACT binds to FhaB on the surface of *B. pertussis* and *B. bronchiseptica* (43), together with the revelation that full-length FhaB plays an important role during infection (11) and that the FhaB prodomain is degraded in a regulated manner (24, 25), suggested a model in which the binding of ACT to its receptor on a phagocytic cell triggers degradation of the FhaB prodomain and delivery of ACT to the host cell (Fig. 1). In this study, we investigated various aspects of the model.

Although previous studies showed that the N-terminal ACD of ACT binds to the mature C-terminal domain of FhaB in both *B. pertussis* and *B. bronchiseptica* (6) and that the binding is strong enough to require molar amounts of urea to remove ACT from FhaB (38, 51–53), the nature of the interaction was not explored further. We showed here that upon secretion, ACT binds FhaB on the bacterial surface before the toxin can be detected in extracellular supernatants (Fig. 2). Efficient binding of ACT by FhaB suggests that the interaction between these proteins is important for the function of one, the other, or both during infection. Our data suggest that for FhaB, the interaction is primarily important for defense against clearance of the bacteria from the lower respiratory tract by phagocytic cells because FhaB mutants in which the prodomain is degraded in an unregulated manner (resulting in increased release of ACT) can adhere to host cells *in vitro* and *in vivo* but are severely defective for persistence in the lower respiratory tract (11). For ACT, we hypothesize that FhaB directs delivery of ACT to phagocytic cells, and not, wastefully, to epithelial cells lacking the ACT receptor or into the extracellular environment.

While the interaction interfaces between FhaB and ACT are not known, binding of the N-terminal ACD of ACT to the FhaB mature C-terminal domain supports the hypothesis that ACT sterically blocks SphB1-dependent cleavage of FhaB (Fig. 4). Our data showing increased amounts of FHA_1/2_ in WCL of the ∆*cyaA* strain compared to WCL of wild-type bacteria (Fig. 3B, WCL) also supports this hypothesis. Previous studies indicate that the ~260 kD FhaB-derived polypeptide in WCL of wild-type *B. bronchiseptica* is FHA′ (the result of DegP- and CtpA-dependent processing) and not FHA (the result of SphB1-dependent cleavage at its primary site on FhaB) (25). FHA is more readily released from the bacterial surface than FHA′ (25), so, given the abundance of FhaB-derived polypeptides in the supernatants of the ∆*cyaA* strain (Fig. 3B, supernatants), it is likely that the larger band is composed of mostly, if not entirely, FHA. Because the formation of FHA requires DegP- and CtpA-dependent degradation of the prodomain (allowing the primary SphB1-dependent cleavage site on FhaB to move through FhaC and become exposed on the cell surface), these data suggest that the presence of ACT also influences DegP- and CtpA-dependent degradation of the prodomain. These data support the hypothesis that when ACT is bound to FhaB, the FhaB prodomain is in a stable conformation that is resistant to DegP-dependent processing (Fig. 1, step 2). In the absence of ACT, or, we propose, when FhaB-associated ACT binds CR3 on host cells, the conformation of the prodomain changes such that DegP cleaves near the FhaB extreme C terminus, exposing a C terminus that is susceptible to processive degradation by CtpA. These data, therefore, provide additional support for the model proposed in Fig. 1.

In this study, we determined that ACT is cleaved at multiple sites in wild-type, but not ∆*sphB1*, bacteria, and we identified the primary cleavage site to be between L325 and T326 (Fig. 7). *Bordetella* strains producing ACT with amino acid substitutions at and near this cleavage site were unable to efficiently intoxicate J774A.1 cells (Fig. 10), most likely due to the proximity of these sites to a loop required for ACT catalytic activity (47). Examination of the site of cleavage also indicates that the ACT^C2^ will not have catalytic activity (48, 54, 55). It is possible that ACT^C2^, while catalytically inert, can form pores in eukaryotic cells. However, the contribution of ACT-dependent pore formation to *Bordetella* pathogenesis has yet to be determined. Like ∆*cyaA* strains, *B. bronchiseptica* strains producing a catalytically inactive form of ACT are rapidly cleared from the murine lower respiratory tract (9), indicating that catalytic activity is the primary function of ACT during infection. Therefore, we interpret the finding that a majority of ACT is converted to catalytically inactive ACT^C2^ in overnight cultures of *B. bronchiseptica* to indicate that SphB1-dependent cleavage of ACT is the result of a degradative process rather than a maturation event, that is, accumulation of the SphB1-dependent ACT^C2^ polypeptide on the cell surface may be an artifact of growing *B. bronchiseptica* for an extended time *in vitro*. Our previous work led to a similar conclusion for SphB1-dependent cleavage of FhaB (25), and hence we propose that SphB1 in *B. bronchiseptica* may function primarily as a “clean-up” protease that removes damaged or “spent” FhaB and ACT proteins from the bacterial surface.

The presence of ACT had a substantial effect on SphB1-dependent degradation of FhaB (especially when the bacteria were cultured in standard SS medium); however, the presence of FhaB had little to no effect on SphB1-dependent cleavage of ACT, indicating that surface retention of ACT by FhaB is not necessary for SphB1-dependent cleavage of ACT (Fig. 3B and 8). Growth in a medium containing 2 mM Ca^2+^ did, however, result in the release of dramatically more full-length ACT into the culture supernatant compared with growth in standard SS (Fig. 3C and 4). These data are consistent with Ca^2+^ facilitating folding and secretion of the RTX domain of ACT (44) and suggest that in standard SS, insufficient Ca^2+^ slows the folding and secretion of ACT such that it is more accessible and more readily cleaved by SphB1.

Although bacteria grown in standard SS or SS containing 2 mM Ca^2+^ retained ACT on the bacterial surface (indicated by dot blot analysis), bacteria grown in SS containing 2 mM Ca^2+^ aggregated and formed a biofilm-like ring at the air-liquid interface of the test tube, suggesting increased FhaB-FhaB interactions and increased FhaB-mediated adherence to glass (Fig. 3C and 4). The amount of biofilm in wild-type bacteria grown in SS containing 2 mM Ca^2+^ was, however, less than the amount of biofilm that occurred in cultures of ∆*cyaA* bacteria grown in either standard SS or SS containing 2 mM Ca^2+^ (Fig. 3C). Together with results from Bumba et al. (44), our data suggest that although 2 mM Ca^2+^ facilitates folding, secretion, and release of ACT from the bacterial surface, a substantial amount of ACT binds FhaB on the surface of *B. bronchiseptica* grown in 2 mM Ca^2+^ (Fig. 3A).

A central feature of our model is the hypothesis that *Bordetella* can deliver FhaB-associated ACT to CR3+ eukaryotic cells. Gray et al. investigated this hypothesis using *B. pertussis* and concluded that it is newly secreted, not FhaB-bound, ACT that is delivered to phagocytic cells (50). We used a different approach, decorating the surface of ∆*cyaA B. bronchiseptica* with ACT from supernatants of a ∆*fhaB* ∆*sphB1* donor strain, and found that these bacteria could cause increased cAMP in J774A.1 macrophage-like cells (Fig. 11). Because the only ACT present in these bacteria was that which was bound to FhaB, our data show definitively that *B. bronchiseptica* can deliver FhaB-associated ACT to host cells. However, the intoxication of host cells by surface-associated (FhaB-bound) ACT or by ACT that has been secreted by its T1SS and avoided FhaB are not mutually exclusive. One possibility is that ACT bound to FhaB serves as a reservoir of active toxin that allows rapid inactivation of patrolling phagocytic cells and that after delivery of the initial surface-associated ACT, the bacteria then continue to deliver more ACT to the host cell either *via* FhaB or directly from the T1SS.

A major challenge of all microbial pathogenesis research is reconciling results obtained from *in vitro* studies with the events that occur during infection. Our analyses have revealed interactions between FhaB, ACT, and SphB1 and are consistent with the model we have proposed in Fig. 1. The fact that *B. bronchiseptica* mutants that produce FhaB proteins that are hyper-processed *in vitro* are severely defective for persistence *in vivo* (11) also supports the model. However, the extent to which these interactions occur *in vivo* will require either new technology that allows us to observe molecular interactions during infection or clever genetic approaches that yield irrefutable results from infection studies, or both. Moreover, for at least three other TPS systems, the C terminus of the TpsA protein is delivered to target cells (other bacteria, in the case of CdiA from *Escherichia coli* (56), host cells in the case of IbpA from *Histophilus somni* (57), and potentially both bacterial and host cells for CdiA from *Pseudomonas aeruginosa* (58)), and hence the possibility that binding to host cells triggers translocation of the FhaB prodomain through FhaC and delivery of the C-terminus to a host cell cannot, at this time, be ruled out.

## MATERIALS AND METHODS

### Culture media and conditions

*Bordetella bronchiseptica* strains were streaked from −80°C stocks onto Bordet-Gengou agar (BD Biosciences) supplemented with 15% defibrinated sheep blood (HemoStat Laboratories) and grown at 37°C for 2–3 days. For broth cultures, colonies were picked from these plates and cultured overnight in Stainer-Scholte broth (SS) (15). For experiments that monitored production and processing of nascent FhaB and ACT across time (such as in Fig. 8), bacteria were instead first grown overnight in BvgAS non-inducing media (SS supplemented with 50 mM MgSO_4_) to prevent the production of Bvg-induced virulence factors, then washed with Dulbecco’s phosphate-buffered saline (PBS; Thermo Fisher), and sub-cultured in fresh BvgAS inducing media (standard SS) for times listed. *Escherichia coli* strains were grown at 37°C in lysogeny broth or on lysogeny broth agar. Where appropriate, media was supplemented with streptomycin (20 µg/mL), kanamycin (50 µg/mL), ampicillin (100 µg/mL), MgSO_4_ (50 mM), and CaCl_2_ (1.8 mM).

### Bacterial strains and mutant creation

Bacterial strains and plasmids are listed in detail in Table S1. *Escherichia coli* strains were used to amplify vectors (DH5α) and to transform plasmids (RHO3) into *B. bronchiseptica*. In-frame deletions in *B. bronchiseptica* were created *via* allelic exchange using derivatives of the pSS4245 plasmid, gene disruptions were created *via* insertion of the pEG7 plasmid within a coding region, and HA tags were added by allelic exchange. Mutations were confirmed by PCR and/or sequencing. As published in the past, we use two strains interchangeably as our wild-type: RB50, recovered from a naturally infected rabbit and RBX11, an RB50 derivative in which *fhaB* is more genetically tractable because the strain lacks *fhaS*, a gene highly homologous to *fhaB,* but that plays no role in virulence (59). Similarly, we often engineered specific mutations across more than one of these lineages. For ease of reading and because we have seen no lineage-dependent differences in regard to FhaB or ACT, we have not specified which lineage was used for specific experiments. Please note that FhaS does appear in culture supernatants when FhaB is absent (RB50 Δ*fhaB*).

### Immunoblots

To examine cell-associated proteins by western blot, whole cell lysates were prepared by boiling pelleted cells in Laemmli buffer. To examine released proteins, culture supernatants were filtered through 0.2 µm filters, and proteins were precipitated using 10% trichloroacetic acid, then rinsed with acetone, resuspended in 1 M Tris-HCl pH 8.8 and Laemmli buffer mixture, and boiled. Proteins were separated by SDS-PAGE using 4% or 5% polyacrylamide gel and transferred to nitrocellulose membranes (GE Healthcare). Membranes were probed with mouse monoclonal antibody generated against HA-tag peptide (YPYDVPDYA; BioLegend), rabbit polyclonal antibody generated against the entire FhaB mature C-terminal domain (FhaB residues 1981 to 2471; (10)), mouse monoclonal antibody that recognizes the AC domain of ACT (3D1; generated using ACT residues 373 to 399 (45); supplied courtesy of F. Heath Damron), mouse monoclonal antibody that recognizes the RTX domain of ACT (9D4; generated using ACT residues 1156 to 1489 (52); supplied courtesy of F. Heath Damron), or rabbit polyclonal antibody generated against the entire ACT protein (46). Corresponding α-rabbit and α-mouse IRDye secondary antibodies (LI-COR Biosciences) were used to detect proteins using a LI-COR Odyssey Classic Blot Imager (LI-COR Biosciences). *B. bronchiseptica* sample volumes were normalized based on the optical density of the cultures.

To examine cell surface exoproteins by dot blot, bacteria were washed with PBS, and 100 µL of 0.5 OD_600_/mL bacterial suspension was spotted onto nitrocellulose membranes using a 96-well vacuum manifold. Membranes were probed using α-HA, α-FhaB, or polyclonal α-ACT antibodies and then by fluorescent secondary antibodies listed above. All blots shown represent at least three biologically independent experiments.

### Edman sequencing to identify SphB1-dependent cleavage site on ACT

The *B. bronchiseptica* RBX9 ∆*batB* strain was grown overnight in SS media supplemented with 25 µM Streptomycin. The supernatants were collected and 110 µL of a 1:1 mixture of culture supernatants and 2× Lamelli buffer were run on 5% SDS-PAGE at 60 mA for 2 hours. The proteins were transferred to PVDF membranes at 250 mA for 16 hours at 4˚C. The membranes were stained with Coomassie Blue stain (5% acetic acid, 30% methanol, and 1 g/L Coomassie R250). The band corresponding to ACT^C2^ was marked, the blot was destained in water to remove Coomassie, and the portion of the membrane containing ACT^C2^ was excised from the membrane and was submitted to the Stanford Protein and Nucleic Acid Facility (PAN) for Edman sequencing of the N-terminal end of the ACT^C2^ polypeptide.

### Eukaryotic cell intoxication assays

J774A.1 mouse-derived macrophage-like cell line was obtained from ATCC. CHO-K1 Chinese hamster ovary epithelial-like cell line and CHO-K1 producing human CR3 were supplied courtesy of Peter Sebo. Eukaryotic cells were grown in Dulbecco’s Modified Eagle Medium with high glucose and pyruvate (Thermo Fisher), supplemented with 10% fetal bovine serum (VWR) and 2 mM L-glutamine (Gibco). J774A.1 cells were additionally supplemented with 1% MEM Non-Essential Amino Acids (Gibco). To examine ACT intoxication of eukaryotic cells, the medium was removed from 6-well plates of eukaryotes and replaced with bacteria-containing growth media. Plates were spun for 5 minutes at 500 × *g* to bring bacteria into contact with the eukaryotic cells, then incubated at 37°C for 15 or 30 minutes. Cells were lysed with 0.1 M HCl with 0.5% Triton X-100 for 20 minutes, and cell debris was pelleted by centrifugation at 21 k × *g* for 10 minutes. Levels of cAMP in the supernatants were determined by competitive ELISA using the manufacturer’s protocol (ENZO). cAMP levels were reported as the ratio of cAMP between infected and uninfected eukaryotic cells: [(pmol/mL cAMP from 10^6^ infected cells)/(pmol/mL cAMP from 10^6^ uninfected cells).

### Surface-associated ACT delivery assays

Recipient strains of *B. bronchiseptica* were unable to produce ACT (∆*cyaA*) or ACT and FhaB (∆*cyaA* ∆*fhaB*) and ACT+ donor strains (∆*fhaB* ∆*sphB1*) were grown overnight in standard SS media. Supernatants were collected from the donor strain cultures and filtered through 0.22 µM filters to remove any bacteria cells. Recipient cells were washed twice in DPBS to remove secreted proteins and, resuspended in the filtered ACT+ donor supernatants and incubated on ice for 30 minutes. The recipient bacteria were spun down onto murine J774A.1 macrophage-like cells at 500 × *g* for 5 minutes at an MOI of 10. Following 30 minutes of infection, the amount of cAMP within the eukaryotic cell lysate was quantified as described above. The data shown in Fig. 11 include results from 16 biologically independent experiments.

### Statistical analysis

We analyzed the results of the cAMP ELISA experiments using two-tailed unpaired *t*-tests using Prism10 (GraphPad) software. We analyzed the results of at least four biologically independent experiments for each data set. Statistical significance was indicated in the figures and figure legends as: **P < 0.01, ***, *P* < 0.001 ****P < 0.0001.

## SUPPLEMENTAL MATERIAL

Supplemental material mbio.00632-24-s0001.pdf Table S1 and Figures S1 to S5.

ASM does not own the copyrights to Supplemental Material that may be linked to, or accessed through, an article. The authors have granted ASM a non-exclusive, world-wide license to publish the Supplemental Material files. Please contact the corresponding author directly for reuse.
