# Supplementary material for: Bordetella filamentous hemagglutinin and adenylate cyclase toxin interactions on the bacterial surface are consistent with FhaB-mediated delivery of ACT to phagocytic cells
Source: mBio. 2024 Mar 27;15(5):e00632-24. doi: 10.1128/mbio.00632-24 (PMC11077949; doi:10.1128/mbio.00632-24)
Supplement: Supplemental material — Table S1 and Figures S1 to S5. [file mbio.00632-24-s0001.pdf]

Supporting Information

Table S1. Bacterial strains and plasmids used in this study

Bacterial strains	Description	Supp. Reference
RB50	*Bordetella bronchiseptica* Complex I strain, isolated from naturally infected rabbit respiratory tract	(1)
RBX11	RB50 containing a deletion of *fhaS* codons 4-3203	(2)
RBX11 HA-PRR	RBX11 containing an insertion of nine codons that encode the HA epitope (YPYDVPDYA) following *fhaB* codon 3375.	(3)
RBX11 Δ*sphB1*	RBX11 containing a deletion of codons 5-1035 of *sphB1*	(4)
RBX9F	RBX9 with a deletion mutation of the *fimA*-*fhaB* intergenic region	(5)
RBX24	RBX9 containing a deletion of *sphB1* codons 5-1035	this work
RB515	RB50 containing a deletion of *cyaA* codons 5-1701	(6)
RB516	RBX9 containing a deletion of *cyaA* codons 5-1701	(6)
RB518	RB50 with deletions of codons 5-1035 of *sphB1* and *cyaA codons 5-*1701	this work
RB512	RB50 producing ACT with an HA epitope (YPYDVPDYA) after the start codon	this work
RB512 Ω*sphB1*	RB512 with plasmid disruption within *sphB1*	this work
RB513	RB50 producing ACT with an HA epitope (YPYDVPDYA) immediately before the stop codon	this work
RB513 Ω*sphB1*	RB513 with plasmid disruption within *sphB1*	this work
RB519	RB50 producing ACT with SQMLTR (aa322-327) mutated to GVIDVE encompassing the SphB1 cleavage site on ACT	this work
RB519 Ω*sphB1*	RB519 with plasmid disruption within *sphB1*	this work
RB522	RB50 producing ACT with M324D and L325P	this work
RB523	RB50 producing ACT with M324Y and L325F	this work
$SphB1^{NT\text{-}HA}$	RB50 producing SphB1 with an HA epitope (YPYDVPDYA) after Pro 58	this work
DH5α	*E. coli* cloning strain	Thermo Fisher
RHO3	*E. coli* conjugation strain and DAP auxotroph	(7)
SM10*λ*pir	*E. coli* conjugation strain	(8)
**Plasmid**	**Description**	**Reference**
pSS4245	Allelic exchange plasmid for *B. bronchiseptica*. Used to create in-frame deletions.	(9)
pCI51	pSS4245 derivative used to insert HA coding sequence in *cyaA* after ATG start con	this work
pCI52	pSS4245 derivative used to insert HA coding sequence in *cyaA* before stop codon	this work
pCI53	pSS4245 derivative used to delete codons 5-1701 in Δ*cyaA*	(6)
pCI55	pSS4245 derivative used to change *cyaA* to encode SQMLTR (aa322-327) to GVIDVE encompassing SphB1 cleavage site on ACT	this work
pCI58	pSS4245 derivative used to change cyaA to encode M324D and L325P	this work
pCI59	pSS4245 derivative used to change cyaA to encode M324Y and L325F	this work
pEG7 Ω*sphB1*	pEG7 derivative used to disrupt the *sphB1* locus	this work

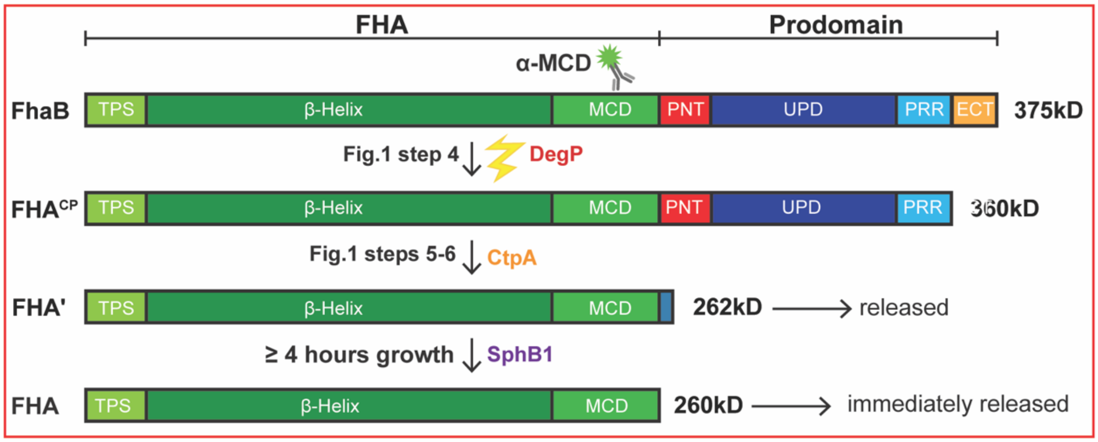

Figure S1. Linear diagram of FhaB processing by multiple proteases.

A signal (lightning bolt), hypothesized to be engagement of the FhaB-ACT complex with receptors on host cells, causes DegP to remove the Extreme C-terminus (ECT) that protects the FhaB prodomain from CtpA (Fig. 1 step 4). This clipped product ($FhaB^{CP}$) is recognized by CtpA, which degrades the majority of the prodomain to form FHA'. CtpA degradation of the PNT allows a portion of FHA' near its C terminus to emerge on the surface (Fig. 1, steps 5-6), which is cleaved by the exoprotease SphB1 to produce FHA (not shown in Fig. 1). FHA is then rapidly released from the bacterial surface.

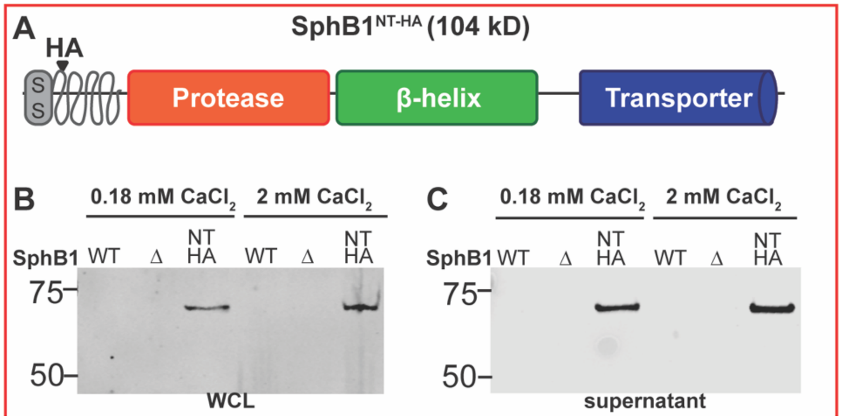

Figure S2. ***B. Bordetella*** **grown in both standard and high calcium produce and secrete similar amounts of SphB1.**

A. Diagram of SphB1^{NT-HA} indicating the domains within SphB1 and the location of the HA insertion following the signal sequence (SS) after proline 58. **B.** WCL and supernatants collected from the SphB1^{NT-HA} strain grown in standard SS (0.18 mM $CaCl_2$) or SS supplemented with high calcium (2 mM $CaCl_2$) contain similar amounts of SphB1^{NT-HA} polypeptides.

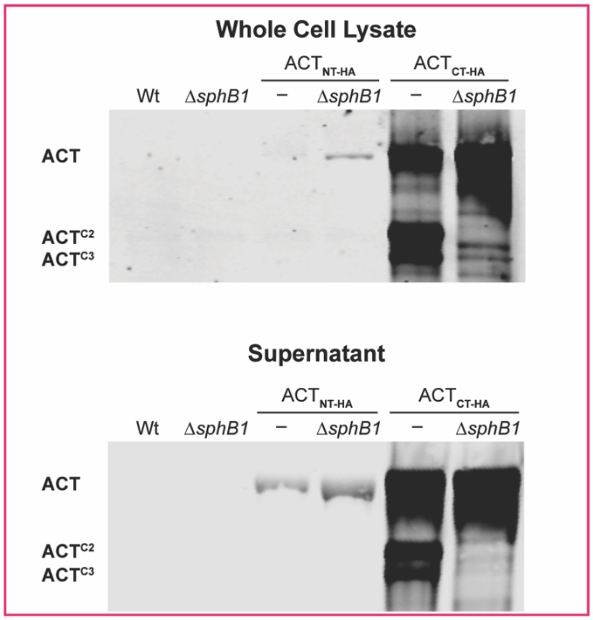

Figure S3. N-terminal HA tag on ACT is readily removed.

Western blots from Figure 6 but showing only HA signal, which has been brightened to show signal in ACT^{NT-HA} samples.

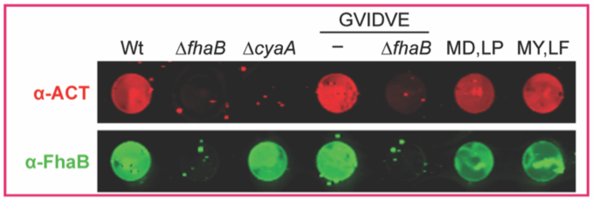

Figure S4. Less cleaved ACT variants still bind FhaB on the bacterial surface.

Surface-exposed ACT and FhaB were labelled on intact wild-type, Δ*fhaB*, Δ*cyaA* bacteria, and also on strains with amino acid substitutions in ACT near the predicted SphB1-dependent cleavage site using polyclonal antibodies generated against the MCD region of FhaB, or all of ACT.

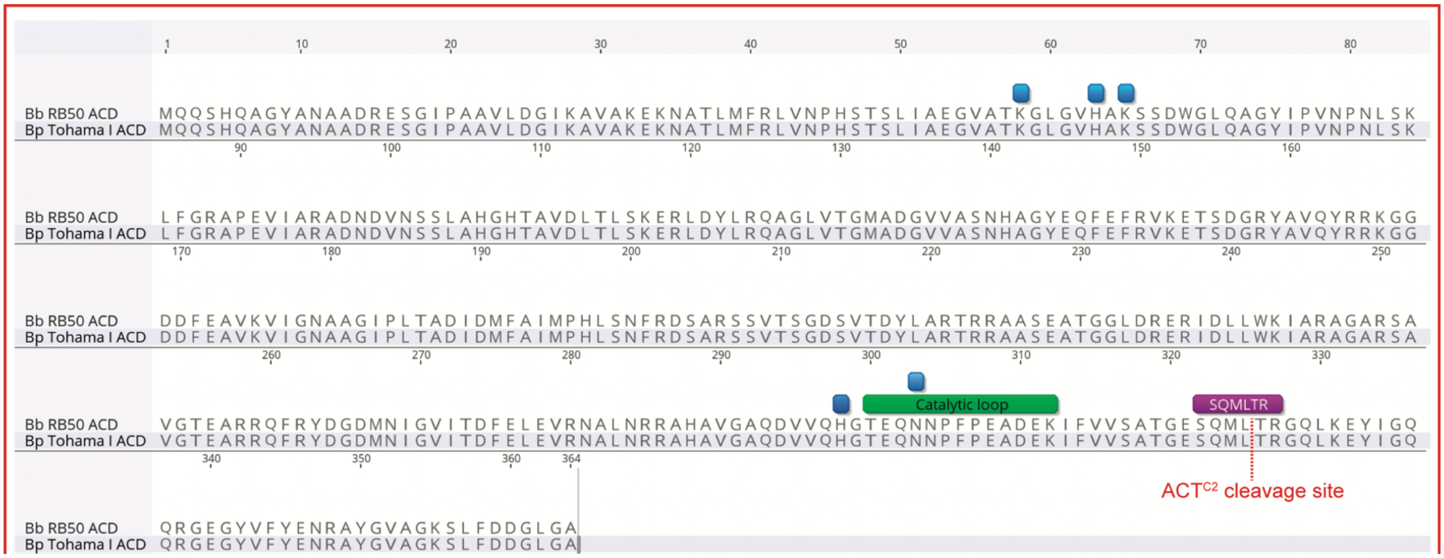

Figure S5. The *B. bronchiseptica* RB50 ACD and the *B. pertussis* Tohama I ACD are identical at the protein level.

Clustal Omega (supplemental ref 10) alignment between the amino acid sequences of the ACT ACDs from *B. bronchiseptica* RB50 and the *B. pertussis* Tohama I. Residues required for catalytic activity (blue), the catalytic loop (green, T300-K312), and the ACT^{C2} cleavage site (dotted red line) are indicated.

Supplemental Bibliography

1. Cotter PA, Miller JF. BvgAS-mediated signal transduction: analysis of phase-locked regulatory mutants of Bordetella bronchiseptica in a rabbit model. Infect Immun. 1994;62(8):3381–90.

2. Julio SM, Cotter PA. Characterization of the Filamentous Hemagglutinin-Like Protein FhaS in Bordetella bronchiseptica. Infect Immun. 2005;73(8):4960–71.

3. Melvin JA, Scheller EV, Noël CR, Cotter PA. New Insight into Filamentous Hemagglutinin Secretion Reveals a Role for Full-Length FhaB in Bordetella Virulence. Mbio. 2015;6(4):e01189-15.

4. Noël CR, Mazar J, Melvin JA, Sexton JA, Cotter PA. The prodomain of the Bordetella two-partner secretion pathway protein FhaB remains intracellular yet affects the conformation of the mature C-terminal domain. Mol Microbiol. 2012;86(4):988–1006.

5. Mason E, Henderson MW, Scheller EV, Byrd MS, Cotter PA. Phenotypic bistability in Bordetella. Mol Microbiol. 2013;90(4):716–33.

6. Henderson MW, Inatsuka CS, Sheets AJ, Williams CL, Benaron DJ, Donato GM, et al. Contribution of Bordetella filamentous hemagglutinin and adenylate cyclase toxin to suppression and evasion of interleukin-17-mediated inflammation. Infect Immun. 2012;80(6):2061–75.

7. López CM, Rholl DA, Trunck LA, Schweizer HP. Versatile Dual-Technology System for Markerless Allele Replacement in Burkholderia pseudomallei ▿ †. Appl Environ Microb. 2009;75(20):6496–503.

8. Miller VL, Mekalanos JJ. A novel suicide vector and its use in construction of insertion mutations: osmoregulation of outer membrane proteins and virulence determinants in Vibrio cholerae requires toxR. J Bacteriol. 1988;170(6):2575–83.

9. Inatsuka CS, Xu Q, Vujkovic-Cvijin I, Wong S, Stibitz S, Miller JF, et al. Pertactin Is Required for Bordetella Species To Resist Neutrophil-Mediated Clearance ▿ . Infect Immun. 2010;78(7):2901–9.

10. Sievers F, Wilm A, Dineen D, Gibson TJ, Karplus K, Li W, et al. Fast, scalable generation of high-quality protein multiple sequence alignments using Clustal Omega. Mol Syst Biol. 2011;7(1):539–539.
